# Underwater wireless communication via TENG-generated Maxwell’s displacement current

**DOI:** 10.1038/s41467-022-31042-8

**Published:** 2022-06-09

**Authors:** Hongfa Zhao, Minyi Xu, Mingrui Shu, Jie An, Wenbo Ding, Xiangyu Liu, Siyuan Wang, Cong Zhao, Hongyong Yu, Hao Wang, Chuan Wang, Xianping Fu, Xinxiang Pan, Guangming Xie, Zhong Lin Wang

**Affiliations:** 1 Marine Engineering College, Dalian Maritime University, 116026 Dalian, China; 2 Tsinghua-Berkeley Shenzhen Institute, Tsinghua Shenzhen International Graduate School, Tsinghua University, 518055 Shenzhen, China; 3 Beijing Institute of Nanoenergy and Nanosystems, Chinese Academy of Sciences, 100085 Beijing, China; 4 Southern Marine Science and Engineering Guangdong Laboratory (Guangzhou), Guangzhou, 511458 P. R. China; 5 College of Engineering, Peking University, Beijing, 100871 P.R. China; 6 School of Materials Science and Engineering, Georgia Institute of Technology, Atlanta, GA 30332-0245 USA

**Keywords:** Sensors and biosensors, Electrical and electronic engineering, Applied physics

## Abstract

Underwater communication is a critical and challenging issue, on account of the complex underwater environment. This study introduces an underwater wireless communication approach via Maxwell’s displacement current generated by a triboelectric nanogenerator. Underwater electric field can be generated through a wire connected to a triboelectric nanogenerator, while current signal can be inducted in an underwater receiver certain distance away. The received current signals are basically immune to disturbances from salinity, turbidity and submerged obstacles. Even after passing through a 100 m long spiral water pipe, the electric signals are not distorted in waveform. By modulating and demodulating the current signals generated by a sound driven triboelectric nanogenerator, texts and images can be transmitted in a water tank at 16 bits/s. An underwater lighting system is operated by the triboelectric nanogenerator-based voice-activated controller wirelessly. This triboelectric nanogenerator-based approach can form the basis for an alternative wireless communication in complex underwater environments.

## Abstract (web summary)

Underwater communication, despite constant development, still remains a challenging technology. Here, authors report an underwater wireless communication approach based on the triboelectric nanogenerator, which provides a self-powered communication system in complex underwater environments.

## Introduction

In the booming ocean exploration, underwater equipment and technology is attracting more and more attention^1–5^. Particularly, obtaining underwater wireless communication has always been a critical challenge. The current underwater communication is achieved through different physical fields, such as acoustic field, optical field, and electromagnetic field^6–8^.

Acoustic communication is most widely used underwater communication as sound wave is not absorbed by water so easily like electromagnetic wave and optical wave. However, acoustic communication has always been accompanied by considerable transmission delays while the transmission is subject to influences from temperature, pressure, and salinity, which leads to multipath effects and Doppler frequency shift. What’s more, echo and reverberation from obstacles could make acoustic communications inaccessible in certain environments (such as confined space, narrow pipes, tunnels, and caves)^9,10^. Underwater optical communication can realize large-capacity data transmission, but it is subject to absorption, scattering, beam divergence, and ambient light interruptions^11,12^. Compared to the acoustic and optical waves, the electromagnetic waves are not affected by acoustic noise or turbulence. Underwater displacement current communication usually has high transmission rate and low delay^10^. While high-frequency electromagnetic waves will be largely absorbed by water^13,14^, low-frequency electromagnetic waves can transmit through an antenna of several kilometers. In sum, complex and sometimes confined underwater space turns out to be a considerable challenge to traditional underwater communication technologies.

Under those conditions, an alternative communication that can work well in underwater space is definitely needed. An inspiration comes to our mind from the Maxwell’s equations, foundation of modern wireless electromagnetic communication. The displacement current, corresponding to ∂**D**/∂*t* in the Maxwell’s equations, is what unified electricity and magnetism theoratically^15^. Of the two terms in displacement current, the first term ∂**E**/∂*t* induces electromagnetic waves widely used in information technology, especially in wireless communications. The second term ∂**P**/∂*t* in the displacement current is induced by the polarization of media^16,17^. Previous studies by Prof. Z. L. Wang reveal that the second term ∂**P**/∂*t* in the Maxwell’s displacement current can be directly related to the output electric current of the nanogenerator^18–25^. A few studies have been performed on the energy transmission or communications (in air), based on the Maxwell’s displacement current generated by the triboelectric nanogenerator (TENG). Recently, Zi et al. (2021) used TENGs to generate a rapidly alternating electric field so that wireless communication in air can be realized by the displacement current ∂**P**/∂*t*^26^. Compared with air, water is of a larger dielectric constant, which is more conducive to the propagation of the polarization electric field. Therefore, based on the second term of the displacement current, i.e., the polarization electric field, underwater communication in complex waters is feasible.

In this study, an underwater wireless communication via Maxwell’s displacement current, ∂**P**/∂*t*, is proposed. The TENG that converts sound to electricity is connected to a transmitting electrode to generate the time-varying polarization electric field underwater. The corresponding time-varying current is measured with a receiving electrode connected to an electrometer. The study reveals that the current signals generated by the TENG yield good anti-interference ability to underwater disturbances. Through a 100 m long salt water pipe, the peak value of the current signal decreases by 66% from the original signal, while the waveforms of the electric signals are not distorted. With the on-off keying method, texts and images can be successfully transmitted in a water tank. No errors appeared in the continuous transmission for about 20,000 digital signals, and an underwater lighting system has been voice-controlled wirelessly via the TENG. What’s more, the current signals output by a sandwich-like TENG can be transmitted in a 50 m × 30 m × 5 m basin with the signals displayed on screen in real time. Therefore, it is believed that the presented work could become an effective communication approach in underwater environments.

## Results

### Working principle of the underwater electric field communication

**Fig. 1:**
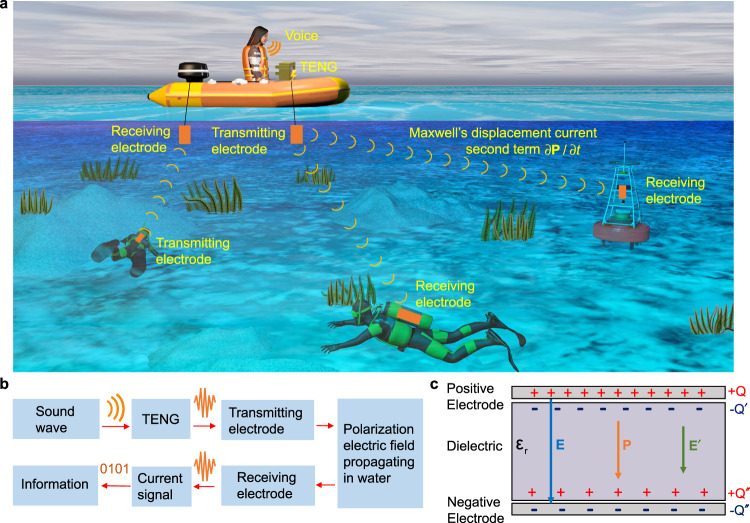
Conceptual diagram of the underwater wireless communication. (signals generated by TENG is directly transmitted without amplification by an external power source). **a** The application and (**b**) the flow chart of the underwater wireless communication. **c** Schematic diagram of the capacitance model, ${{{\varepsilon }}}_{{{r}}}$ is relative permittivity, **E** is the original electric field, **P** is the polarization electric field, **E′** is the combined electric field of (**E**) and (**P**), and all about Q are amount of charge.

A conceptual diagram of the application of the studied underwater communication is shown in Fig. 1. The TENG converts sound (i.e. sonic waves in air) to electric signals in water (Fig. 1a), and the electric signals carrying the voice information can be transmitted in water and received by the diver. In this way, an underwater wireless communication is established via Maxwell’s displacement current generated by the TENG. Figure 1b is the flow chart of the underwater communication. It needs to be noted that this method is different from the electric field communication generated from a pair of electric dipoles (see Supplementary Note 1).

The working principle of the underwater communication can be understood, approximately, with a capacitance model (Fig. 1c). The propagation of underwater electric field is analyzed from the perspective of displacement current. The transmitting and receiving electrodes form the positive and negative electrodes of the capacitor, while the water solution is the dielectric. With the presence of electric field **E**, the dielectric can be polarized where a polarization electric field **P** can be generated. It should be noted that the polarization electric field **P** is originated from the negative polarization charge to the positive polarization charge. ${{{{{\bf{E}}}}}}^{\prime}$ is the combined electric field of **E** and **P**, If the relative permittivity is defined as ${\varepsilon }_{r}={{{{{\bf{E}}}}}}{{{{{\boldsymbol{/}}}}}}{{{{{\bf{E}}}}}}^{\prime}$, the relationship between the polarization charge and the charge *Q* on the transmitting electrode is ${Q}^{\prime} =(1-1/{\varepsilon }_{r})Q$. Due to the attenuation of the electric field during through propagation medium, the received charge $Q^{\prime\prime}$ at the receiving electrode is <$Q^{\prime}$.

The underwater communication can be demonstrated theoretically with the Maxwell’s equation. Remind that the Gauss’s law of the Maxwell’s equations is

1 $$\nabla \cdot {{{{{\bf{D}}}}}}={{{{{\boldsymbol{\rho }}}}}}$$

where ρ is the distribution of free charges in space, and **D** is the electric displacement vector, which can be expressed as

2 $${{{{{\bf{D}}}}}}={{{\varepsilon }}}_{{{0}}}{{{{{\bf{E}}}}}}+{{{{{\bf{P}}}}}}$$

where permittivity in vacuum is ${{{\varepsilon }}}_{{{0}}}$. The Maxwell’s displacement current density can be defined as

3 $${{{{{{\bf{J}}}}}}}_{{{{{{\bf{D}}}}}}}=\frac{\partial {{{{{\bf{D}}}}}}}{\partial {t}}={{{\varepsilon }}}_{{{0}}}\frac{\partial {{{{{\bf{E}}}}}}}{\partial {t}}+\frac{\partial {{{{{\bf{P}}}}}}}{\partial {t}}$$

From Eq. (3), the first term ${{{\varepsilon }}}_{{{0}}}\partial {{{{{\bf{E}}}}}}/\partial {t}$ gives rise to electromagnetic wave. Studies of Prof. Zhonglin Wang reveal that the second term (∂**P**/∂t) in the Maxwell’s displacement current can be directly related to the output electric current of the TENG^15^.

It is worth mentioning that the internal circuit in the TENG is dominated by the displacement current, and the observed current in the external circuit is the capacitive conduction current (see Supplementary Note 2). The research on the underwater electric field propagation is inspired by the built-in electric field of the TENG. Comparing the TENG-based underwater electric field with electromagnetic waves, the propagation of electromagnetic waves does not require a medium, and the propagation effect is best in vacuum. At this time, ∂**E**/∂t reaches the maximum value, and ∂**P**/∂t is 0. The propagation of the polarization electric field requires a medium (see Supplementary Note 3). As ∂**E**/∂t gets significantly reduced in water, the propagation effect of ∂**P**/∂t gets improved.

**Fig. 2:**
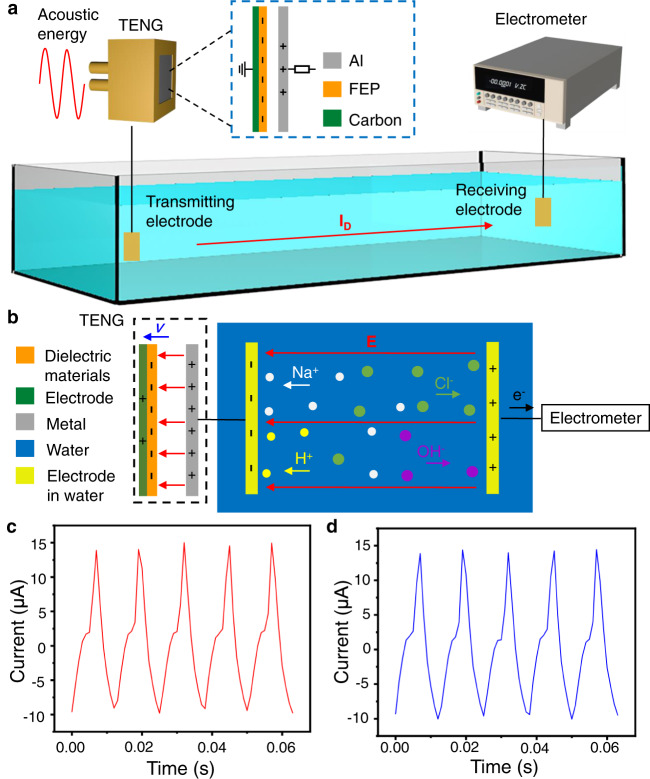
The electric signals of an acoustic-driven TENG in air and water. **a** Schematic diagram of the experimental process. *I*_D_ represents displacement current in all figures. **b** Schematic diagram of the working principle. **E** is the underwater electric field, and *v* is the speed of the TENG for contact and separation. **c** The short-circuit current signals (measured by connecting an electrometer to the aluminum electrode). **d** The short-circuit current signal obtained by connecting the electrometer to the receiving electrode.

To examine the performance of the underwater communication, an acoustic-driven TENG is applied to convert sound in air to electrical signals in water. The output performance of the acoustic-driven TENG has been investigated systematically in our previous study^27^. As shown in Fig. 2a, the TENG consists of a Helmholtz resonant cavity, an aluminum film with evenly distributed acoustic holes, and a fluorinated ethylene propylene (FEP) film with a conductive ink-printed electrode (details about the TENG is shown in Supplementary Notes 4 and 5). The transmitting electrode in water is connected to the aluminum electrode of the TENG, while the other electrode of the TENG is grounded so that the TENG operates in the single-electrode mode. In reaching an electrostatic equilibrium state, higher electrical output can be obtained by acquiring ground charges^28^. It is worth noting that one piece of conduct materials, such as metal and salt water, can serve as a charge reservoir for the TENG.

Figure 2b shows the working principle of the underwater communication, which is based on the interface polarization from the Maxwell-Wagner effect. The electrical output is generated from the variation of the built-in electric field in the TENG, which is directly related to the second term (∂**P**/∂t) in the Maxwell’s displacement current^15^. A transmitting electrode is connected to one electrode of the TENG, thus an electric field ${{{{{{\bf{E}}}}}}}_{{{{{{\bf{0}}}}}}}$ is induced in water as the TENG works (see Fig. 2b). Corresponding to the variation of electric field ${{{{{{\bf{E}}}}}}}_{{{{{{\bf{0}}}}}}}$, the positive and negative ions in the water move reciprocally, generating a polarization electric field ${{{{{{\bf{P}}}}}}}_{{{{{{\bf{0}}}}}}}$. The current in the receiving electrode induced by the polarization electric field can be measured with an electrometer. The electric field ${{{{{{\bf{E}}}}}}}_{{{{{{\bf{0}}}}}}}$ generated by the TENG is related to the charge density ${{{{{{\boldsymbol{\rho }}}}}}}_{{{{{{\boldsymbol{0}}}}}}}$ in the transmitting electrode in the following form:

4 $$\nabla \cdot ({{{\varepsilon }}}_{{{0}}}{{{{{{\bf{E}}}}}}}_{{{{{{\bf{0}}}}}}}+{{{{{{\bf{P}}}}}}}_{{{{{{\bf{0}}}}}}})={{{{{{\boldsymbol{\rho }}}}}}}_{{{{{{\boldsymbol{0}}}}}}}$$

${{{{{{\bf{P}}}}}}}_{{{{{{\bf{0}}}}}}}$ is the polarization electric field generated from the electric field ${{{{{{\bf{E}}}}}}}_{{{{{{\bf{0}}}}}}}$, which is

5 $${{{{{{\bf{P}}}}}}}_{{{{{{\bf{0}}}}}}}=\frac{{{{\varepsilon }}}_{{{r}}}-1}{{{{\varepsilon }}}_{{{r}}}}{{{\varepsilon }}}_{{{0}}}{{{{{{\bf{E}}}}}}}_{{{{{{\bf{0}}}}}}}$$

Therefore, the second term ${{{{{{\bf{J}}}}}}}_{{{{{{\bf{p}}}}}}}$ in the Maxwell’s displacement current (generated by the polarization electric field) is

6 $${{{{{{\bf{J}}}}}}}_{{{{{{\boldsymbol{\rho }}}}}}}=\frac{\partial {{{{{{\bf{P}}}}}}}_{{{{{{\boldsymbol{0}}}}}}}}{\partial {t}}=\frac{{{{\varepsilon }}}_{{{r}}}{{{{{\boldsymbol{-}}}}}}1}{{{{\varepsilon }}}_{{{r}}}}{{{\varepsilon }}}_{{{0}}}\frac{\partial {{{{{{\bf{E}}}}}}}_{{{{{{\bf{0}}}}}}}}{\partial {t}}$$

For the acoustic driven HR-TENG, when the FEP film contacts with the aluminum film, the electron clouds on the surfaces of the two films overlap, and some of the electrons from the aluminum film enter the deeper potential well of the FEP film. Due to the much higher electronegativity of FEP than aluminum, the free electrons on the surface of the aluminum film transfer to the lowest unoccupied molecular orbital of the FEP interface. So the aluminum film becomes positively charged (Supplementary Fig. 1a). Since the transmitting electrode is connected to the aluminum film, positive charges are also distributed on the surface of the transmitting electrode. Negatively charged ions in the water are attracted by the transmitting electrode, while positively charged ions are repelled to the surroundings. When positive ions contact with the receiving electrode, electrons in the receiving circuit flow to the receiving electrode, so the electrometer detects a positive current. Due to the change in the acoustic pressure difference, the FEP film is separated from the aluminum electrode. At the moment, electrons flow from the ground to the conductive ink electrode to balance the electric field between the FEP film and the conductive ink electrode. Due to the negative charge distributed on the surface of the FEP film, the free electrons on the aluminum film are repelled, so the electrons flow from the aluminum film to the transmitting electrode. Opposite to before, positive charged ions in the water are attracted by the transmitting electrode, while negative charged ions are repelled to the surroundings (Supplementary Fig. 1b). When negative ions contact with the receiving electrode, electrons in the receiving electrode flow to the receiving circuit, so the electrometer detects a negative current.

Figure 2c, d compares the electric signals in air with those in ordinary water. Under acoustic waves (80 Hz, 80 dB), the corresponding periodic output short-circuit current signals yield the peak value of 14.9 μA (Fig. 2b), which is directly measured with the electrometer connected to the aluminum electrode. When the (electrometer-connected) receiving electrode is two meters away from the submerged transmitting electrode, the peak value of the current decreases slightly to 14.5 μA while the waveform of electric signals remain constant (Fig. 2c). The peak value of open-circuit voltage output of the TENG decreases from 28.5 V in air to 13 V in water (see Supplementary Fig. 2). When the water tank is grounded by a wire, the output current decreases significantly, but the waveform of electric signals stay consistent with the original signal (Supplementary Fig. 3). This can be explained by the tendency that charges from ground would balance the electrical potential field in water. Furthermore, the current signal could still be measured even when the transmitting electrode is insulated from water by a Kapton tape (Supplementary Fig. 4), and the electric field generated by the TENG can propagate across both gas and liquid media (Supplementary Fig. 5). These prove that the transmission of the signals depends on the electric field radiated by the TENG rather than the direct exchange of electrons between water and electrode plates. Both theoretical analysis and experiments have shown that for the whole system, the propagation of underwater electric field has demonstrated the characteristic of displacement current (see Supplementary Note 6). Previous study^29^ proved that when the electric field propagates in a medium, conduction current dominates when ${{{{{\boldsymbol{\sigma }}}}}}{{{{{\boldsymbol{/}}}}}}{{{{{\boldsymbol{\omega }}}}}}\; > \;{{\varepsilon }}$ and displacement current dominates when ${{\varepsilon }}\, > \,{{{{{\boldsymbol{\sigma }}}}}}{{{{{\boldsymbol{/}}}}}}{{{{{\boldsymbol{\omega }}}}}}$ (σ is conductivity, ω is angular frequency, and ε is permittivity). The Rayleigh distance of the TENG generated electric field can be calculated by ${{{{{\boldsymbol{R}}}}}}=2{{{{{{\boldsymbol{D}}}}}}}^{{{{{{\boldsymbol{2}}}}}}}{{{{{\boldsymbol{/}}}}}}{{{{{\boldsymbol{\lambda }}}}}}$, where R is Rayleigh distance, D is the maximum size of the transmitting electrode, and λ is the wave length. As a result, R is so small that it can be neglected. According to these theories, for the TENG-based underwater electric field communication, ${{\varepsilon }}\, > \,{{{{{\boldsymbol{\sigma }}}}}}{{{{{\boldsymbol{/}}}}}}{{{{{\boldsymbol{\omega }}}}}}$ and the transmitting distance is larger than the Rayleigh distance. So the displacement current domains the underwater electric field while conduction current only appears in a very short distance.

It is worth mentioning that underwater communication can also be realized by various types of TENGs, such as the TENGs that harvest wave energy and vibration energy (see Supplementary Fig. 6, and the first and second items in Supplementary Table 1). The development of the electric field communication depends on the development of TENG technology. The techniques for designing TENGs with high frequency and good output performance (see Supplementary Table 1) provides good potential for the application of the TENG in underwater communication^30–37^.

### Transmission performance of the underwater electric field

**Fig. 3:**
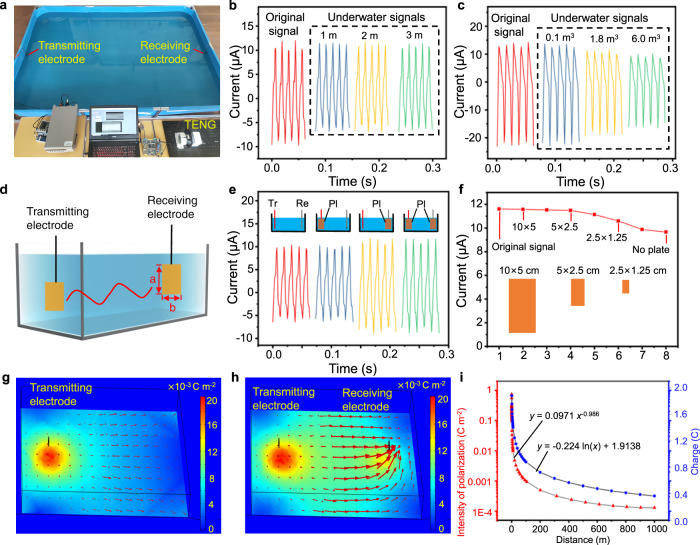
The transmission performance of the underwater electric field. **a** A photo of the experiment. **b** Comparison between the original TENG short-circuit current and received current signals in water. **c** Comparison between the original short-circuit current of the TENG and received signals under different water volume. **d** Schematic diagram of the electrode. **e** Comparison of the current signals received in water with different transmitting and receiving electrodes. Tr, Re, and Pl represents transmitting electrode, receiving electrode, and electrode plate respectively. **f** Comparison of the peak values of the received current signals with different receiving electrodes. Simulation diagram of the distribution of polarization electric field (**g**) without and (**h**) with the receiving electrode. Color represents polarization intensity. **i** Variation of polarization electric field and charges on the receiving electrode with the distance between the two electrodes.

The characteristics of the underwater electric field under different parameters such as water volume, electrode position/size, salinity, water turbidity, and underwater obstacles, have been studied. Figure 3 shows the attenuation of the underwater electric field. In a 3 m × 2 m × 0.4 m water tank (Fig. 3a), the receiving electrode is placed certain distance away from the transmitting electrode. As the distance increases from 1 m to 3 m, the current signals remain almost unchanged (Fig. 3b). This is also verified by the simulation of underwater polarization electric field shown in Supplementary Fig. 7. Furthermore, the complete signals output by the TENG and received signals underwater, and their Fourier transforms are shown in Supplementary Figs. 8–9, showing that the frequency-domain features of the signals remain unchanged underwater. As more water is added into the tank, the peak value of the current decreases. Actually, the peak value decreases by 30% from the original signals as the water volume reaches 6 m^3^ (Fig. 3c). This can be understood, as the electric field energy W in water is

7 $${{{{{\boldsymbol{W}}}}}}=\int_{{{{{{\boldsymbol{V}}}}}}}\frac{{{{{{\boldsymbol{1}}}}}}}{{{{{{\boldsymbol{2}}}}}}}{{{\varepsilon }}}_{{{0}}}{{{\varepsilon }}}_{{{r}}}{{{{{{\boldsymbol{E}}}}}}}_{{{{{{\boldsymbol{0}}}}}}}^{{{{{{\boldsymbol{2}}}}}}}{{{{{\boldsymbol{d}}}}}}{{{{{\boldsymbol{V}}}}}}$$

According to Eq. (7), the energy density of the electric field decreases with the water volume V while using a TENG with constant power output. Therefore, the peak value of the current decreases with larger water volume.

As shown in Fig. 3d–f, the current can be enhanced by using a receiving electrode plate of larger area. With a 10 cm × 5 cm electrode plate, the peak value of the current signal increases by 18%, compared to that with a thin electric wire. This means more ions in water contacting with the electrode, which is verified by the underwater polarization electric field simulation shown in Supplementary Fig. 10. In addition, the peak value of the current is subject to the angle between the transmitting and receiving electrode plates, but the effect caused by the angle is very small in the water tank (see Supplementary Figs. 11–12), which is quite different from the case in a bipolar electric field^38^.

By comparing the distribution of the polarization electric fields without and with the receiving electrode (Fig. 3g, h), it is found that the receiving electrode can change the distribution of the polarization electric field. This can be explained by the fact that the electrode is equivalent to a terminal with a potential of zero voltage. The variation of the polarization electric field and the variation of the terminal charges in the receiving electrode are shown in Fig. 3i. A 2D simulation is performed and the polarization electric field distribution corresponding to Fig. 3i is shown in Supplementary Figs. 13 and 14. The attenuation of the polarization electric field and terminal charges at the receiving electrode with distance can be fitted respectively

9 $${P}={k}_{1}{{x}}^{a}$$

10 $${{Q}}_{{r}}={k}_{2}ln{x}+{k}_{3}$$

*k*_1_, *k*_2_, *k*_3_ and a are parameters in the fitted curves of the output power of the TENG (Supplementary Figs. 15 and 16). According to the simulation results, the exponent of *x* is close to −1 for two dimension simulations (Supplementary Figs. 15 and 16), and close to −2 for three dimension simulations (Supplementary Figs. 17 and 18), which is consistence with the Gauss’s law.

**Fig. 4:**
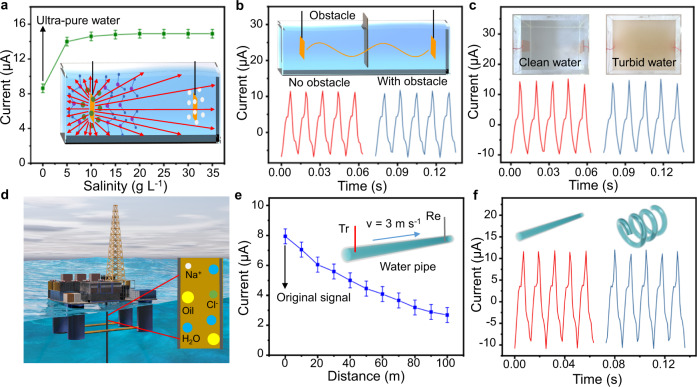
Effects of disturbances on the underwater electric field transmission. **a** Effect of water salinity on the peak value of the current. Error bars indicate standard deviations, with all values ≤0.53. **b** Influence of an obstacle on the current signals. **c** Comparison between the received current signals in clean water and those in turbid water. **d** Schematic diagram of the drilling platform with oil pipeline. **e** Variation of the peak value of short-circuit current output transmitted in a water pipe of 100 meters. Tr and Re represent transmitting and receiving electrodes respectively. *v* is the liquid flowing speed. Error bars indicate standard deviations, with all values ≤0.46. **f** Comparison between the short-circuit current signals transmitted in a straight and those in a curved water pipe.

The dependence of the underwater electric field on disturbances in water is shown in Fig. 4. It is found that the peak value of the current signal in salty water (5 g L^−1^, adjusted by adding salt to water) increases by 40% on top of that in pure water (Fig. 4a). This indicates that the ions in salt water can enhance the polarization electric field. However, water salinity increase beyond 15 g L^−1^ will not further promote the current signals. Similarly, when acid or alkali is added to the pure water to change the pH, the ion concentration in the water will change, indicating that as the pH of water deviates from 7, the received current signals will increase (Supplementary Fig. 19). This may be attributed to the improved relative permittivity of the aqueous solution. Figure 4b, c reveal that the waveform of the received signals identical to original ones, regardless of obstacles or turbidity in the water tank. In this sense, the polarization electric field has shown robustness to obstacles and water turbidity.

As achieving reliable communication across the pipe is very important for the pipeline robot system^39,40^, the performance of the polarization electric field in liquid pipes is investigated (Fig. 4d–f). Figure 4d is a schematic diagram of the drilling platform with the oil pipeline. In a pipe filled with salt water, the peak value of the current is also found to decreases with the distance between the transmitting electrode and receiving electrode. In fact, the value decreases by 66% when the distance in-between is 100 m (Fig. 4e), which is consistent with the simulation result of the polarization electric field in the pipe (as shown in Supplementary Fig. 20).

In fact, the collision between ions and water molecules may influence the performance of the electric field. In addition, it is interesting to find that the received signals in a spiral pipe are the same with those in a straight pipe (Fig. 4f). From Supplementary Fig. 21, it is found that independent of the flow status, the current signals can also be obtained in the mixture of oil and water, which means the polarization electric field communication can be applied in complex pipelines.

It is worth noting that the electric field communication is also insensitive to water temperature and ambient lightness (Supplementary Fig. 22). Further comparisons between acoustic, optical, and electromagnetic waves methods are shown in Supplementary Table 2. What’s more, by studying the effect of the ground on the electric field, it is theoretically proved that this system may work in open water area as shown in Supplementary Note 7.

### Modulation and demodulation of the underwater electric field communication

**Fig. 5:**
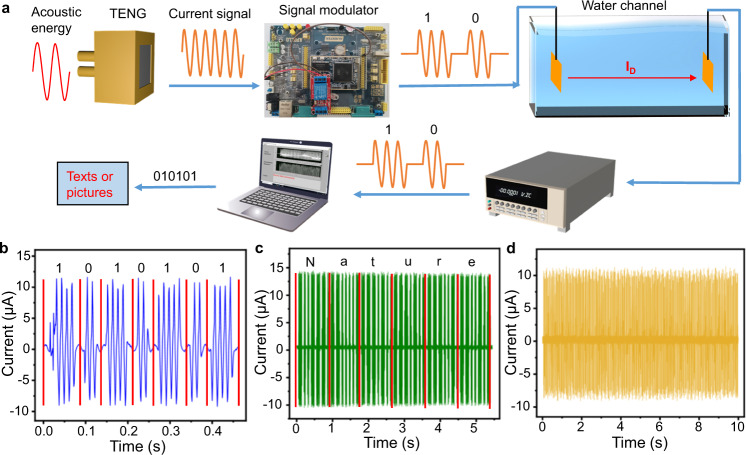
The modulation and demodulation of current signals for data transmission in water. **a** Schematic diagram of the modulation and demodulation process. **b** The modulated digital signals transmitted in water. **c** The demodulated current signals to a word after transmitting in water. **d** Part of current signals transmitted for an image.

The modulation and demodulation process of current signals for data transmission in water is shown in Fig. 5. The current signals converted from sound waves by the TENG can be modulated to digital signals containing the information of texts or images in water via the electric field communication (Fig. 5a). The signal modulation method is based on the on-off keying (OOK), in which longer signals with time intervals of 50 ms is set as “1”, and shorter signals with time intervals of 25 ms is set as “0”. A 25 ms interval is inserted between each digital signal to separate adjacent digital signals. After transmission in water, the modulated digital signals can be received by the electrometer (Fig. 5b). The fundamental frequency of the signals generated by the TENG is 80 Hz, and the frequency of the modulated digital signals is 16 Hz. Alternatively, the digital signals can be modulated with other frequencies or other methods (see Supplementary Fig. 23a, b). Higher frequency yields a fast information transmission rate, while lower frequency yields a strong anti-interference ability.

By demodulating the received signal (with the MATLAB codes), the signals of “0” and “1” can be identified accurately (Fig. 5b and Supplementary Fig. 23c). The current signals can be modulated into text by the standard encoding. The received signals can be accurately demodulated into the original text (Fig. 5c). Supplementary Movie 1 shows that the real-time current signals generated by the TENG is modulated, transmitted, and demodulated, and the text obtained after demodulation is displayed on a computer screen. This electric field communication can also be used for image transmission. Figure 5d shows part of the received current signals, and the complete signals are shown in Supplementary Fig. 23d. A 2.7-KB image is transmitted within 1353 s at 16 bits/s (owing to the low fundamental frequency of the TENG). There is no error signal in the continuous transmission of ~20,000 digital signals (100,000 working cycles of the TENG). In addition, by applying an external alternating current on the dielectric material’s electrode, the electric signals with higher frequencies (up to kilohertz) can be modulated and transmitted in water, demonstrating that this approach can be used for high frequencies communication (see Supplementary Note 8).

It should be noted that the current signals output by the TENG and received signals underwater at a range of 60–200 Hz are compared. It turns out that the signals received underwater are always consistent in waveforms with the signals output by the TENG (see Supplementary Fig. 24). What’s more, the power spectrum is obtained by performing Fourier transform to the modulated digital signals and noise. The power spectrum shows that energy is evenly distributed in the frequency range from 40 kHz to 85 kHz (see Supplementary Fig. 25), proving that the bandwidth of the system with water channel is greater than 85 kHz.

### Realization of wireless control using the underwater electric field communication

**Fig. 6:**
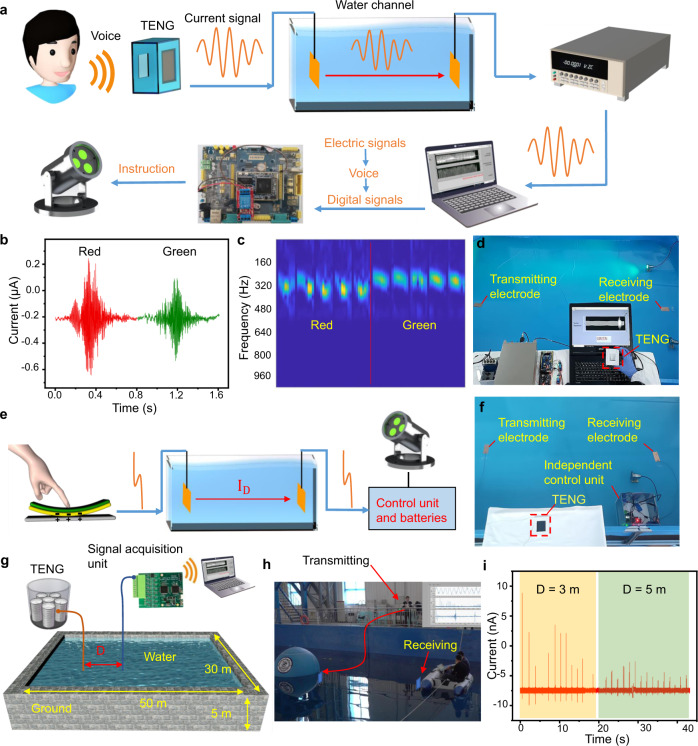
Self-powered communication based on the TENGs for underwater wirelessly controlled system. **a** The schematic diagram of underwater light wirelessly controlled by voice. **b** The received signals of “red” and “green”. **c** The short-time Fourier transform of “red” and “green”. Colors represent amplitudes. **d** The photo of the voice control experiment setup. **e** The experiment of the button-type TENG controlling an independent system. **f** The photo of the touch control experiment. **g** The schematic diagram and (**h**) The photo of the experiment in a 50 m × 30 m × 5 m basin. *D* is the distance between two electrodes. **i** The received current signals underwater.

To further study the ability of the underwater wireless communication, a demo voice control of an underwater lighting system is performed (Fig. 6). A microphone-style TENG that converts voice to electrical signals is to control the underwater lighting system wirelessly (Fig. 6a). The signals containing the voice information (e.g. “red” and “green”) are transmitted in water and received by the electrometer (Fig. 6b). By performing short-time Fourier transform to the signals, the words “red” and “green” can be distinguished with a neural network algorithm (see Fig. 6c and Supplementary Fig. 26). Subsequently, the words are converted into digital signals to control the lights. This approach can be applied in the real-time voice control of underwater lights (Fig. 6d and Supplementary Movie 2). It is worth mentioning that the entire underwater communication realized by the TENG is self-powered.

At the same time, the signals receiving and controlling device in the water can be designed independently. By touching a (contact-separation mode) button-type TENG, people can use the electric to control the independent working system in water (see Fig. 6e, f and Supplementary Movie 3). The independent working system consists of a weak current acquisition board, a single-chip microcomputer, batteries, a relay, and an underwater working light. The pulse signals generated by the TENG are collected by the weak current acquisition board, and the analog signals are converted into digital signals sent to the microcontroller. The single-chip microcomputer processed the digital signals and controlled the underwater working light.

In another demo experiment, a sandwich-like TENG (S-TENG) with an output current of 60 μA is deployed in a 50 m × 30 m × 5 m basin (with all boundaries connected to the ground, see Fig. 6g, h. When the S-TENG is shaken, the current signals outputted by the S-TENG can be transmitted in water and received by the receiving electrode 5 m away from the transmitting electrode. The signals are detected by a current acquisition board (Supplementary Note 9), which sends the signals to a computer through WiFi and then the waveforms are displayed on the screen (Fig. 6i and Supplementary Movie 4).

## Discussion

In summary, an underwater communication via Maxwell’s displacement current is proposed and investigated. In the Maxwell’s displacement current, the first term ∂**E**/∂*t* gives rise to electromagnetic waves. However, in underwater environments, the high-frequency electromagnetic waves can be easily absorbed, and the low-frequency electromagnetic waves can only be transmitted through an antenna of several kilometers. In this study, the second term (∂**P**/∂*t*) in the Maxwell’s displacement current is utilized for underwater communication. An acoustic-driven TENG connected to a transmitting electrode is applied to generate alternating electric field in water, so that the sound in air can be converted into underwater electrical signals, which can be measured with a receiving electrode connected to an electrometer. Through a salt water pipe of 100 m length, the peak value of the current signal decreases by 66% compared to the original signal, while the electric signals are not distorted in waveform during transmission.

Based on the on-off keying method, texts and images have been successfully transmitted by modulated current signals in a water tank at 16 bits/s. Throughout the continuous transmission of about 20,000 digital signals, no error appears. By successfully converting voices into current signals, the TENG is capable of controlling an underwater lighting system wirelessly. What’s more, the current signals output by a sandwich-like TENG can be transmitted in a 50 m × 30 m × 5 m basin with the signals displayed on screen in real time. Compared to traditional sonic, optical, and electromagnetic communications, the underwater communication via Maxwell’s displacement current appears to be less vulnerable to disturbances, which exhibits considerable potential for applications in complex underwater environments.

## Methods

### Fabrication of the TENGs

The HR-TENG in the experiments consists of a Helmholtz resonant cavity, an aluminum film with acoustic holes, and an FEP film with a conductive ink-printed electrode. The resonant cavity has a dimension of 73 mm × 73 mm × 40 mm. Two tubes with an inner diameter of 5.0 mm and a length of 32 mm are fixed on the resonant cavity. The aluminum film with 440 uniformly distributed acoustic holes acts as the electropositive triboelectric layer. The length, width, and thickness of the film are 45 mm, 45 mm, and 0.1 mm, respectively and the diameter of the holes is 0.5 mm. The FEP film is used as the electronegative triboelectric layer on observation of its strong electronegativity and good flexibility. It has a thickness of 12.5 μm and a working area of 45 mm × 45 mm. Given that the FEP material is insulated, a conductive ink electrode with a micron thickness is attached to the other side of the FEP film to transfer electrons. A screen printing device (Tou) is used to print the conductive ink (CH-8(MOD2)) on the FEP film (WitLan). The shell is printed by a 3D printer with PLA material.

The TENG to recognize voice is similar to the HR-TENG, except that it has no dule-tube structure but has a 45 mm × 45 mm opening on one side of the resonance cavity. The contact separation distance between the FEP and aluminum film is ~0.2 mm. The membrane structure of the button-type TENG is the same as HR-TENG without a cavity.

The acrylic plate of a single layer S-TENG is of 5 mm thickness and 10 cm diameter. Two aluminum electrodes with a thickness of 50 µm and an area of 6 cm × 4 cm are parallel attached onto two sides of the acrylic plate. PTFE balls with 10.5 mm diameter are filled between two acrylic plates and they are produced by 3 M company. Each S-TENG unit consists of 10 layers stacked S-TENG in parallel connection and acrylic block shell. The acrylic block shell has 10 cm diameter and 20 cm height. There are four AC output copper ends in an S-TENG unit, one pair at the top and the other pair at the bottom. The buoy consists of 5 S-TENG units as the power module and an acrylic shell as the frame structure. The S- TENG units integrated inside are in parallel connection to make the AC electrical output in-phase and the they are fixed through packing tape.

### Experimental process and measuring equipment

The output signals are measured with a Keithley 6514 electrometer. The HR-TENG is mounted on an optical plate with a loudspeaker (JBL), driven by sinusoidal waves from a function generator (YE1311). One electrode of the TENG is grounded and the other electrode is immersed in water. The wires used for electrodes has a 0.3 mm copper core, and the electrode plates are copper films with the size of 100 × 50 × 0.06 mm. The water pipe used in the experiment is an ordinary PVC rubber water pipe with an inner diameter of 13 mm. A 12 V DC motor is used to control the flow of liquid in the pipe. The signal modulator consists of a microcontroller development board (STM32F7) and a relay. The MATLAB interface in LABVIEW is used to demodulate and display the real-time signals measured with the electrometer. The signals generated by the voice-driven TENG need to be filtered at 50 Hz and its harmonics after being received.

The transmitting electrode in water and the aluminum electrode of the TENG is connected by an ordinary copper wire. The HR-TENG is applied to convert sound in air to electrical signals in water. In this way, acoustic waves with specific frequencies can be got by controlling the signal generator and a loudspeaker. Furthermore, under the excitation of the acoustic waves, electrical signals with specific frequencies can be generated by the HR-TENG to examine the performance of the underwater communication. Button type TENG is a basic and commonly used TENG with the simplest structure. The application of the button type TENG prove that underwater communication can be realized by general TENGs, demonstrating the application potential of this approach.

### Numerical simulations

In order to verify the accuracy of the derived theory and experimental results, COMSOL Multiphysics software has been used for numerical simulations. The AD/DC modules, electrostatic interfaces, and transient state analysis are used in simulations. The distribution of the polarization electric field and terminal charge of the receiving electrode has been simulated. As the element size influences the calculation results, the ultra-fineness meshing option has been adopted in the simulation. For 3-D simulations, the size of the model is limited to 15 m. The 2-D simulation is used to analyze the attenuation of the electric field through longer distance. The error of the 3D simulation depends on the mesh size and model scale.

## Supplementary information

Supplementary Information

Description of Additional Supplementary Files

Supplementary Movie 1

Supplementary Movie 2

Supplementary Movie 3

Supplementary Movie 4

## Data Availability

The data supporting this study are available within the article and the Supporting Information. Source data are provided with this paper.

## Source data

Source Data
