# Supplementary material for: Underwater wireless communication via TENG-generated Maxwell’s displacement current
Source: Nat Commun. 2022 Jun 9;13:3325. doi: 10.1038/s41467-022-31042-8 (PMC9184604; doi:10.1038/s41467-022-31042-8)
Supplement: Supplementary file 1 — Supplementary Information [file 41467_2022_31042_MOESM1_ESM.pdf]

Supplementary information for

Underwater Wireless Communication via TENG-Generated Maxwell's Displacement Current

Hongfa Zhao[1,2,7] Minyi Xu[1,7*], Mingrui Shu[1,7], Jie An[3], Wenbo Ding[2], Xiangyu Liu[1], Siyuang Wang[1], Cong Zhao[1], Hongyong Yu[1], Hao Wang[1], Chuan Wang[1], Xianping Fu[1,4], Xinxiang Pan[1], Guangming Xie[1,4,5*], Zhong Lin Wang[3,6*]

[1] Marine Engineering College, Dalian Maritime University, Dalian, 116026, China

[2] Tsinghua-Berkeley Shenzhen Institute, Tsinghua Shenzhen International Graduate School, Tsinghua University, Shenzhen, 518055, China

[3] Beijing Institute of Nanoenergy and Nanosystems, Chinese Academy of Sciences, Beijing 100085, China

[4] Peng Cheng Laboratory, Shenzhen, 518000, P. R. China

[5] College of Engineering, Peking University, Beijing, 100871, P.R. China

[6] School of Materials Science and Engineering, Georgia Institute of Technology, Atlanta, Georgia 30332-0245, United States

[7] These authors contributed equally: Hongfa Zhao, Minyi Xu, Mingrui Shu

*Email: xuminyi@dlmu.edu.cn; xiegming@pku.edu.cn; zlwang@gatech.edu

Table of contents:

Supplementary Fig. 1 Working mechanism of the underwater electric field with respect to the effect of the HR-TENG. 5

Supplementary Fig. 2 Comparison between the open-circuit voltage output of the TENG and the voltage signal received underwater..6

Supplementary Fig. 3 Electric signals received in water when the water is grounded. 7

Supplementary Fig. 4 Electric signals received in water when the transmitting electrode is insulated. 8

Supplementary Fig. 5 Signals transmission across air and water. a Schematic diagram of the experiment. 9

Supplementary Fig. 6 Realizing underwater communication by different types of TENGs. 10

Supplementary Fig. 7 Variation of the polarization electric field distribution with the distance between two electrodes. 11

Supplementary Fig. 8 Comparison between the original TENG short-circuit current and received current signals in water. 12

Supplementary Fig. 9 Comparison between the Fourier transforms of the original TENG current signals and the received current signals in water. 13

Supplementary Fig. 10 Simulation of the underwater polarization electric field with different electrodes. 14

Supplementary Fig. 11 Simulation diagram of the distribution of polarization electric field with different angles between the transmitting and receiving electrode 15

Supplementary Fig. 12 The current signals received underwater in different angles between the transmitting and receiving electrode plates 16

Supplementary Fig. 14 Simulation of the underwater polarization electric field with two electrodes. 18

Supplementary Fig. 15 Effect of the water volume on the polarization electric field distribution. 19

Supplementary Fig. 16 Effect of the electrode plates area and the charges in the transmitting electrode on the terminal charges. 20
Supplementary Fig. 17 Distribution of the polarization electric field in a water area of 10 × 10 × 10 m. 21
Supplementary Fig. 18 Simulation of the polarization electric field and terminal charges at the receiving electrode in a water area of 10 × 10 × 10 m. 22
Supplementary Fig. 19 Effect of the PH on the peak value of the current. 23
Supplementary Fig. 20 Distribution of the polarization electric field in the water pipe. 24
Supplementary Fig. 21 Short-circuit current output of the TENG and received signals in water. 25
Supplementary Fig. 22 The received current signals under different temperatures and light conditions..26
Supplementary Fig. 23 The modulation current signals for data transmission in water. 27
Supplementary Fig. 24 Received current signals in different frequencies. 28
Supplementary Fig. 25 Power spectrum of the modulated digital signals and noise in water 29
Supplementary Fig. 26 Fourier transform of “red” and “green”. a Fourier transform of “red”. b Fourier transform of “green” 30
Supplementary Table 1. Output performance of different types of TENGs.............31
Supplementary Table 2. Comparison of acoustic, optical, electromagnetic waves and electric field in underwater communication 32
Supplementary Note 1. The difference between traditional and TENG based underwater communication approaches...33
Supplementary Note 2. The working principle of the TENG via Maxwell's displacement current 34
Supplementary Note 3. The experiment in vacuum 36
Supplementary Note 4. The structure of the HR-TENG.......................................37

Supplementary Note 5. Working mechanism of the sound-driven HR-TENG....38
Supplementary Note 6. The analysis of theories and experiments for the whole system..........40
Supplementary Note 7. The effect of the ground on the underwater electric field communication..........42
Supplementary Note 8. The TENG-based underwater communication in high frequencies..........46
Supplementary Note 9. The working principle of the auxiliary circuit................48
Supplementary References..........49

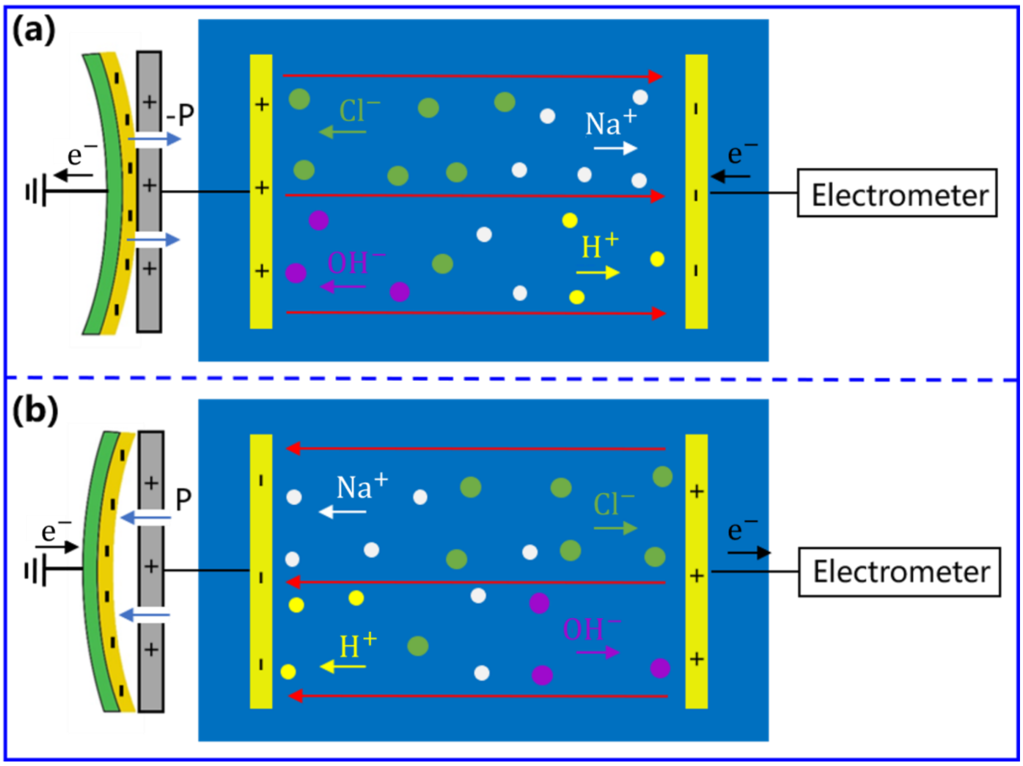

Supplementary Fig. 1 Working mechanism of the underwater electric field with respect to the effect of the HR-TENG. Working mechanism of the underwater electric field when the FEP film **a** contact and **b** separate with the aluminum electrode.

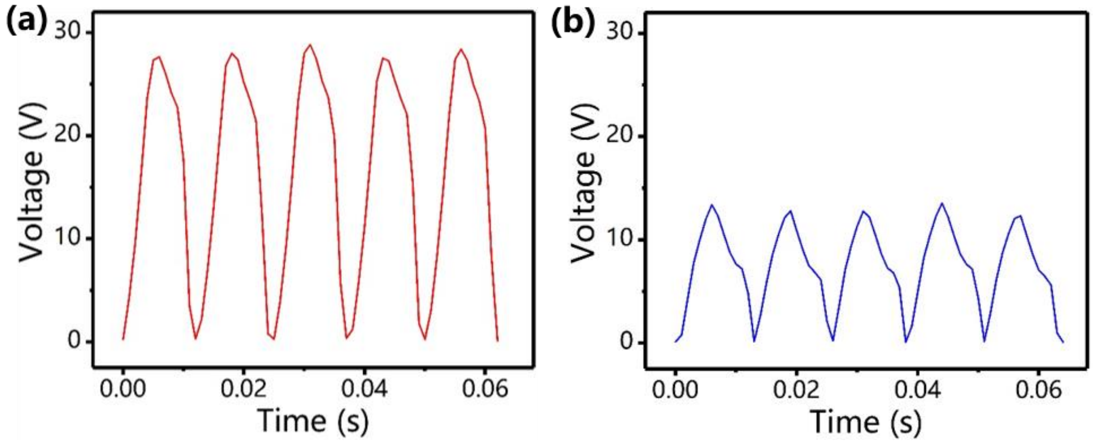

Supplementary Fig. 2 Comparison between the open-circuit voltage output of the TENG and the voltage signal received underwater. **a** The open-circuit voltage output of the TENG. **b** The voltage signal received by the receiving electrode in water.

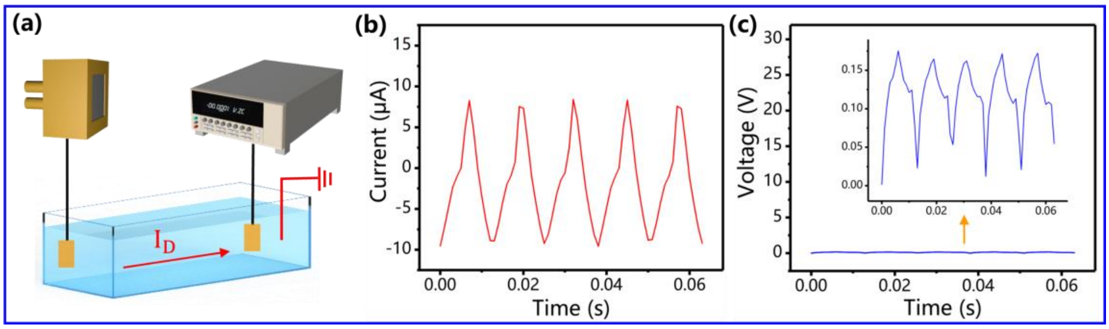

Supplementary Fig. 3 Electric signals received in water when the water is grounded. **a** Schematic diagram of the experiment progress. I_D represents displacement current in all figures. **b** The current signal received in the water. **c** The voltage signal received in the water.

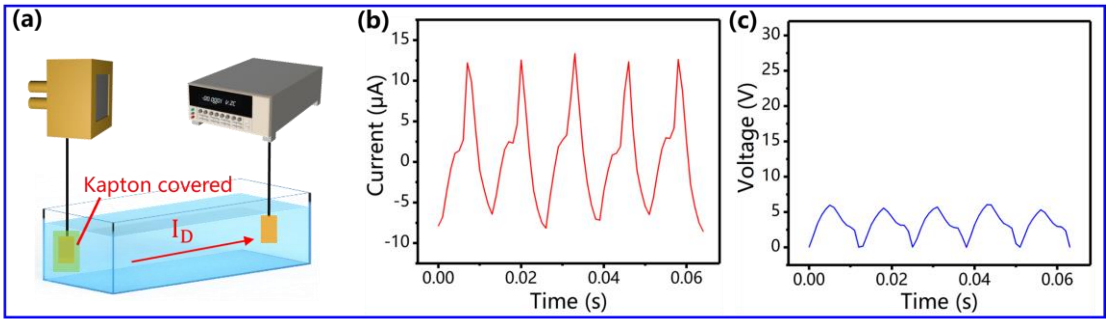

Supplementary Fig. 4 Electric signals received in water when the transmitting electrode is insulated. **a** Schematic diagram of the experiment progress. **b** The current signal received in the water. **c** The voltage signal received in the water.

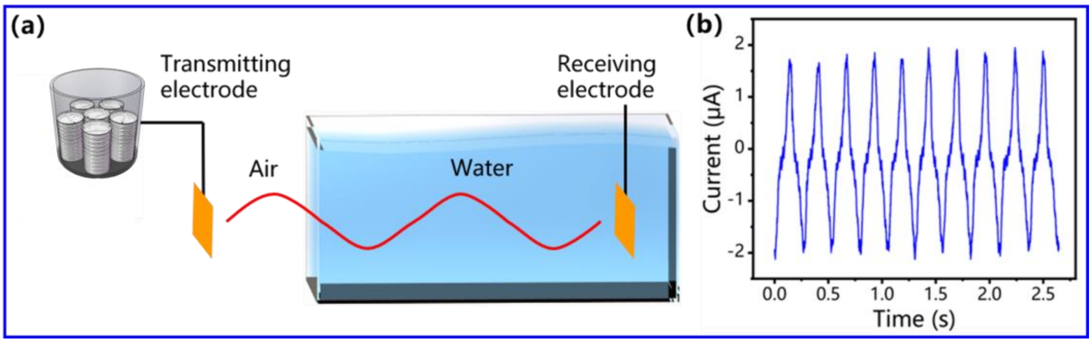

Supplementary Fig. 5 Signals transmission across air and water. a Schematic diagram of the experiment. **b** The underwater current signals received from the receiving electrode.

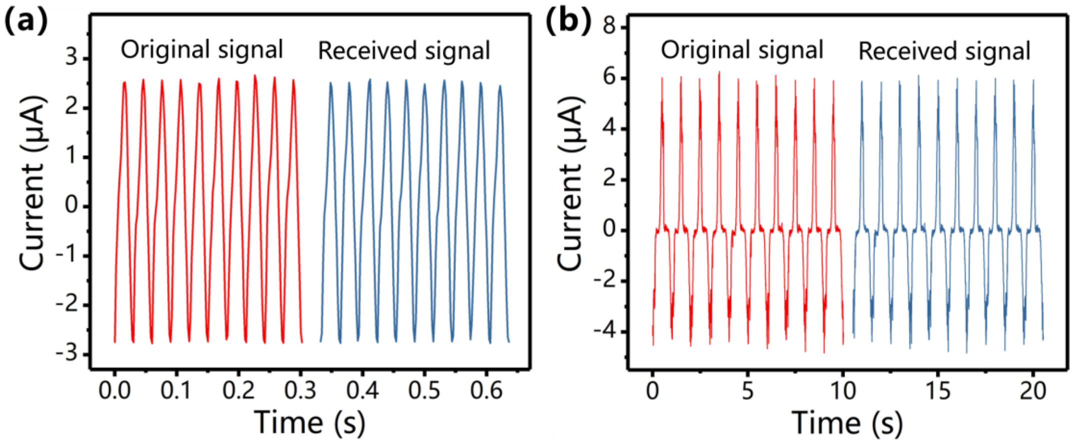

Supplementary Fig. 6 Realizing underwater communication by different types of TENGs. (a) Comparison between the current signals output by the tower-like TENG and current signals received underwater. (b) Comparison between the current signals output by the honeycomb structure inspired TENG and current signals received underwater.

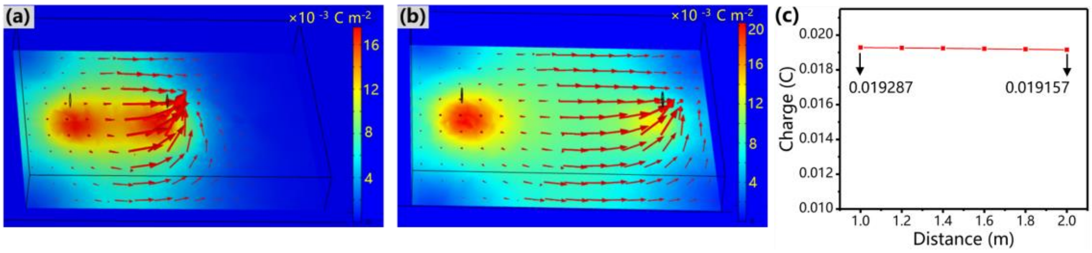

Supplementary Fig. 7 Variation of the polarization electric field distribution with the distance between two electrodes. Color represents polarization intensity, including other simulation figures. **a** Distribution of the polarization electric field when the distance between two electrodes is 2 m **b** Distribution of the polarization electric field when the distance between two electrodes is 1 m. **c** Variation of charges on the receiving electrode with the distance between the two electrodes.

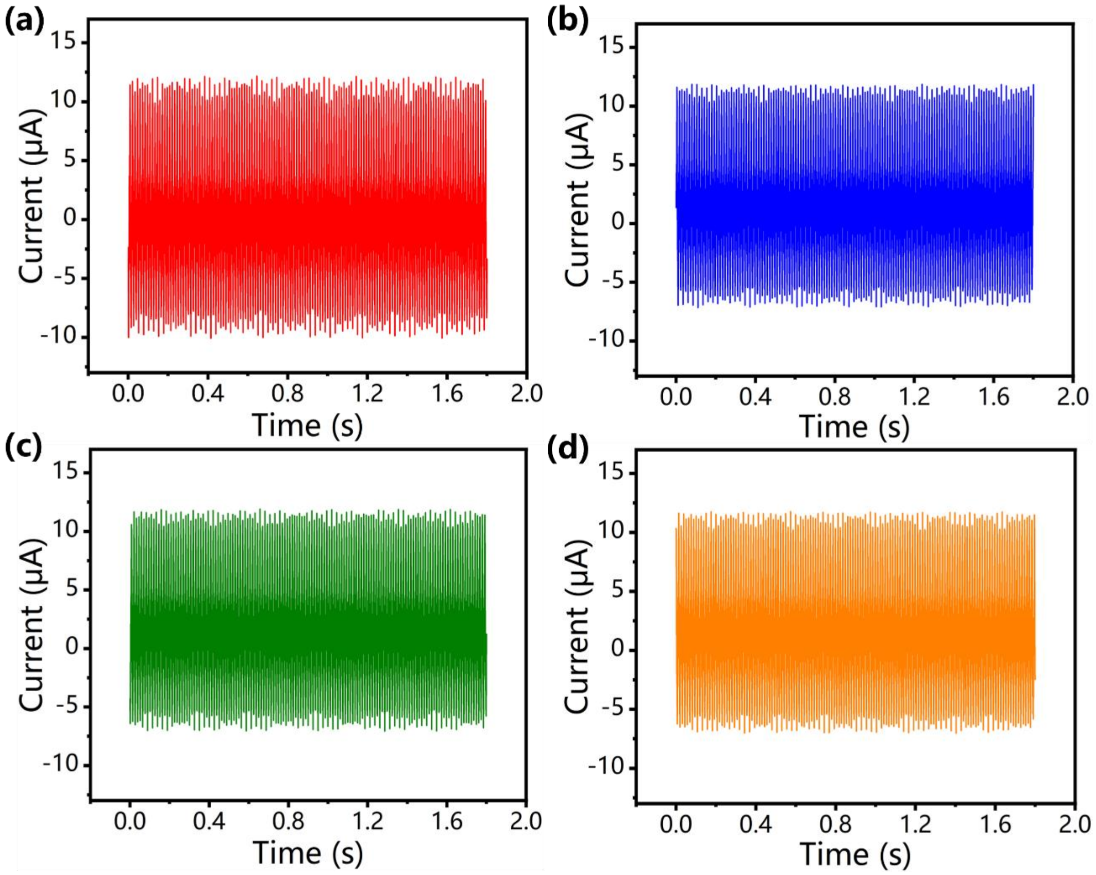

Supplementary Fig. 8 Comparison between the original TENG short-circuit current and received current signals in water. a The current signals output by the TENG. The received current signals underwater when the distances between the transmitting and receiving electrodes are b 1 m, c 2 m and d 3 m.

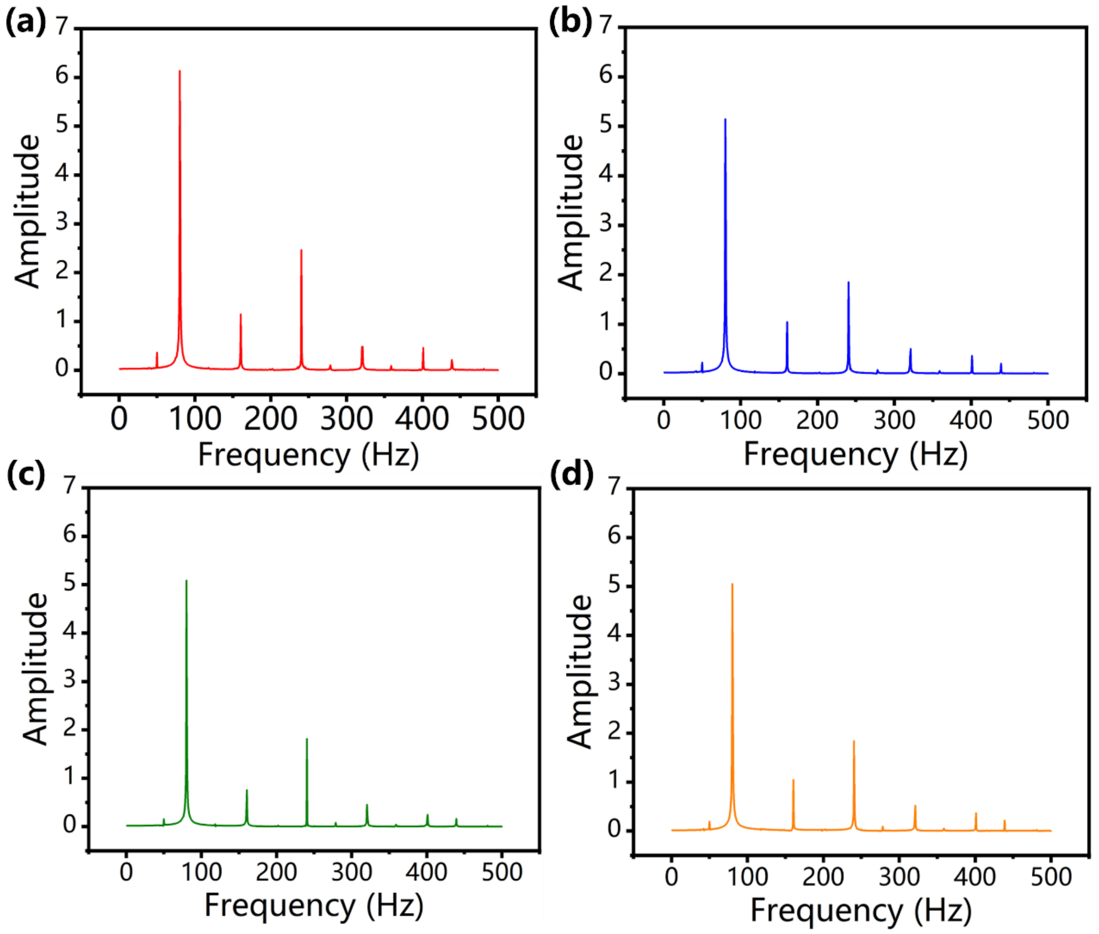

Supplementary Fig. 9 Comparison between the Fourier transforms of the original TENG current signals and the received current signals in water. a Fourier transforms of the current signals output by the TENG. Fourier transforms of the received current signals underwater when the distances between the transmitting and receiving electrodes are b 1 m, c 2 m and d 3 m.

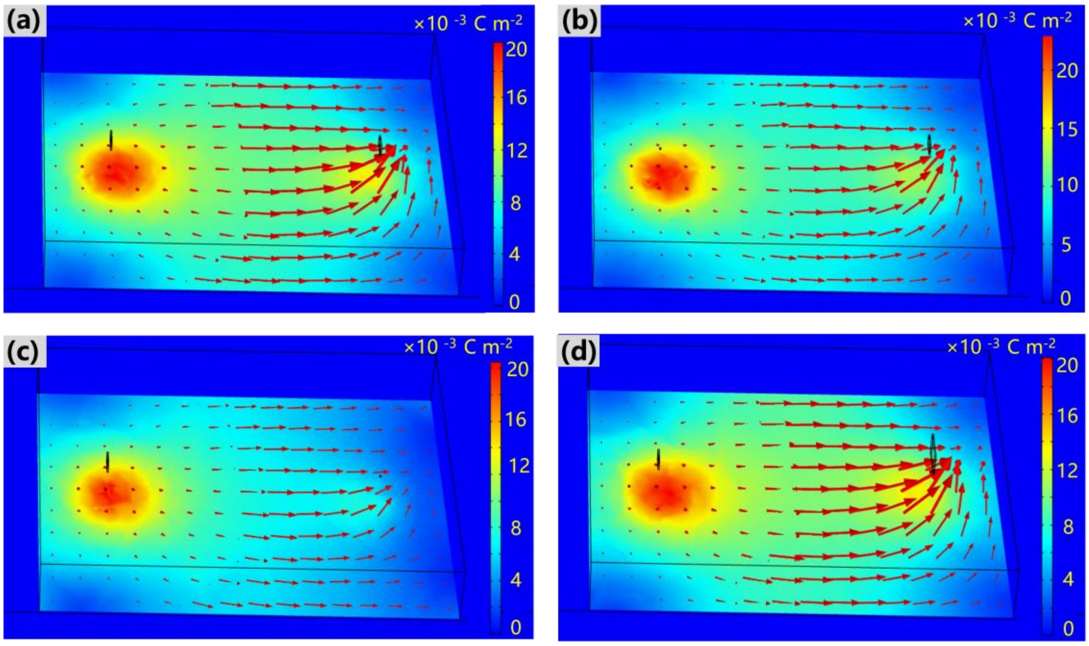

Supplementary Fig. 10 Simulation of the underwater polarization electric field with different electrodes. Distribution of underwater polarization electric field in the case of **a** two electrode plates with the size of 0.1 × 0.1 m; **b** the transmitting electrode plate with a size of 0.01 × 0.01 m and the receiving electrode plate with a size of 0.1 × 0.1 m; **c** the transmitting electrode plate with a size of 0.1 × 0.1 m and the receiving electrode plate with a size of 0.01 × 0.01 m; **d** the transmitting electrode plate with a size of 0.1 × 0.1 m and the receiving electrode plate with a size of 0.2 × 0.2 m.

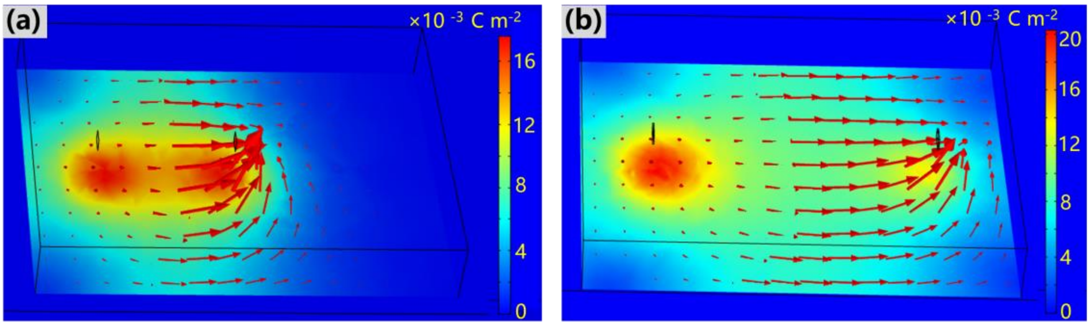

Supplementary Fig. 11 Simulation diagram of the distribution of polarization electric field with different angles between the transmitting and receiving electrode.

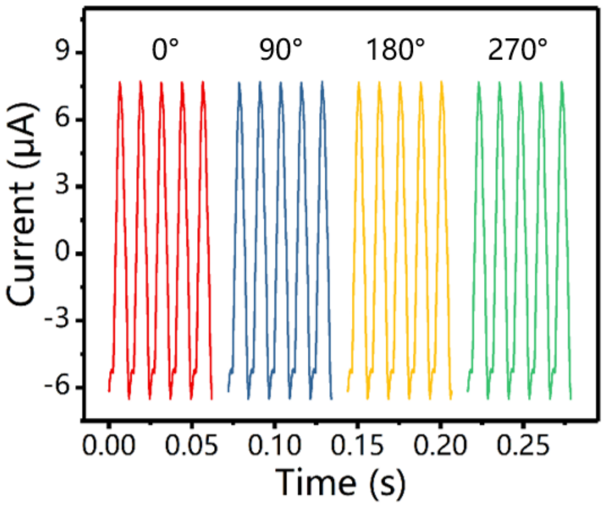

Supplementary Fig. 12 The current signals received underwater in different angles between the transmitting and receiving electrode plates.

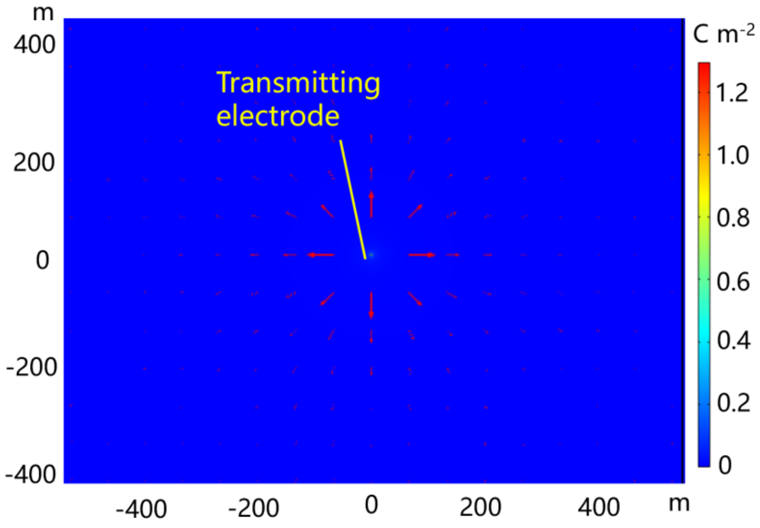

Supplementary Fig. 13 Simulation of the underwater polarization electric field without the receiving electrode.

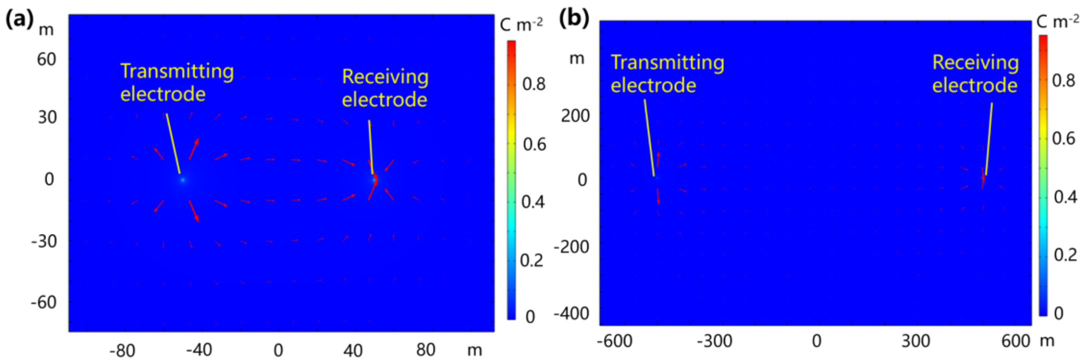

Supplementary Fig. 14 Simulation of the underwater polarization electric field with two electrodes. Distribution of the polarization electric field when the distance between two electrodes is **a** 100 m and **b** 1000 m.

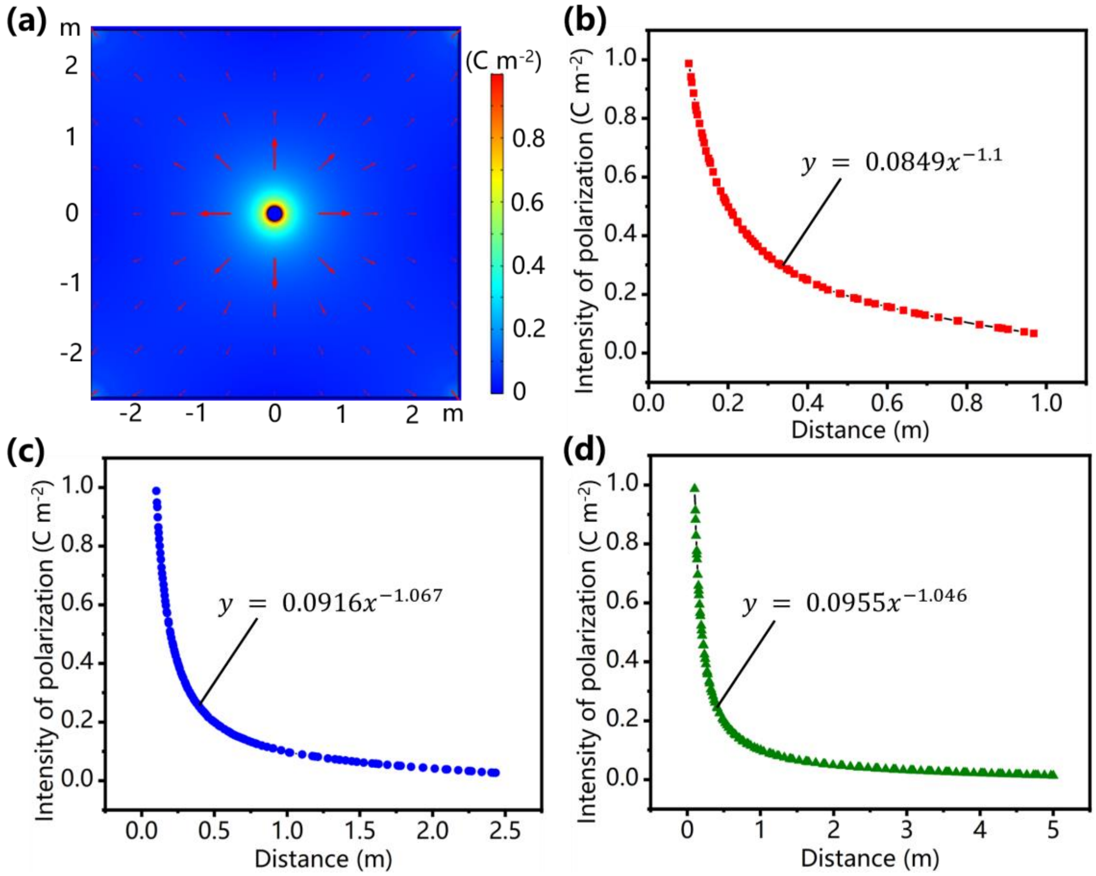

Supplementary Fig. 15 Effect of the water volume on the polarization electric field distribution. **a** Distribution of underwater polarization electric field in a water area of 2.5 × 2.5 m. Variation of the polarization electric field with distance when the water volume are **b** 2 × 2 m **c** 5 × 5 m and **d** 10 × 10 m.

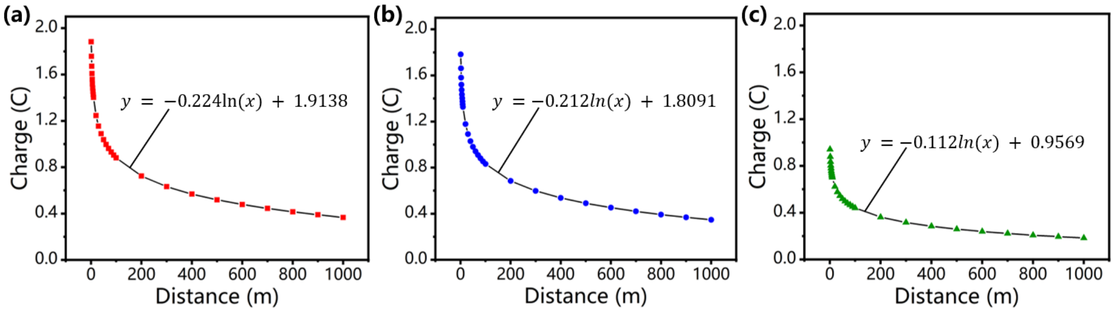

Supplementary Fig. 16 Effect of the electrode plates area and the charges in the transmitting electrode on the terminal charges. The variation of the terminal charges in the receiving electrode with distance when **a** the two electrode plates with the size of 0.1 × 1 m, and 0.2 C charges in in the transmitting electrode; **b** the size of receiving electrode is changed to 0.1 × 0.5 m; **c** the charges in the transmitting electrode decrease to 0.1 C.

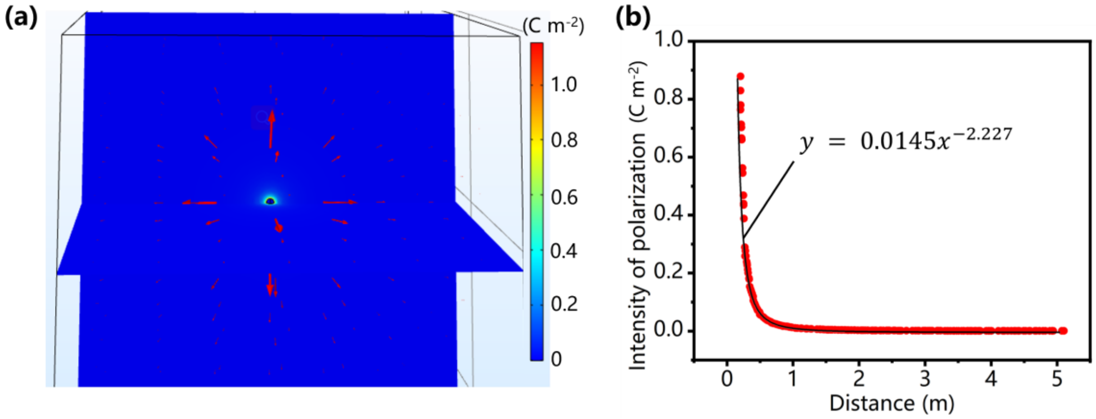

Supplementary Fig. 17 Distribution of the polarization electric field in a water area of 10 × 10 × 10 m. **a** Distribution of underwater polarization electric field. **b** Variation of the polarization electric field with distance.

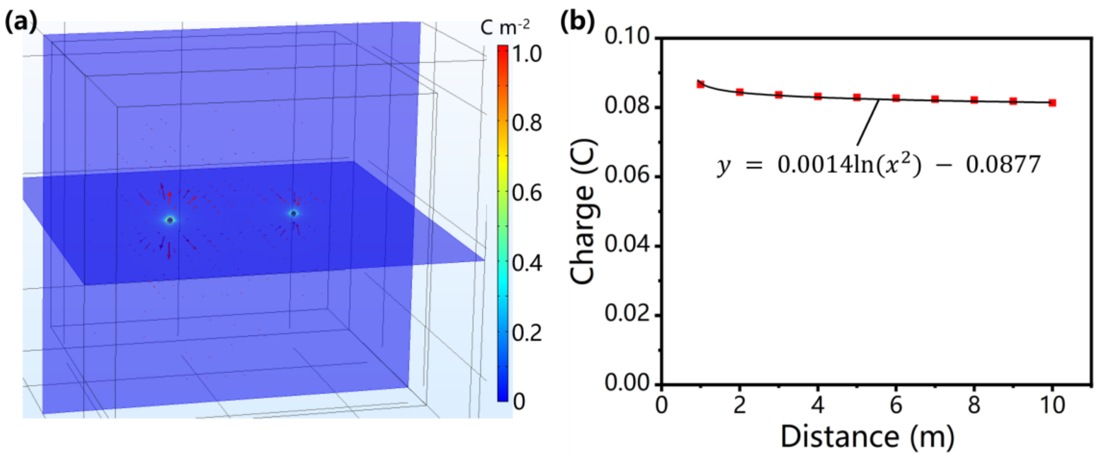

Supplementary Fig. 18 Simulation of the polarization electric field and terminal charges at the receiving electrode in a water area of 10 × 10 × 10 m. **a** Distribution of underwater polarization electric field. **b** Variation of the terminal charges at the receiving electrode with distance.

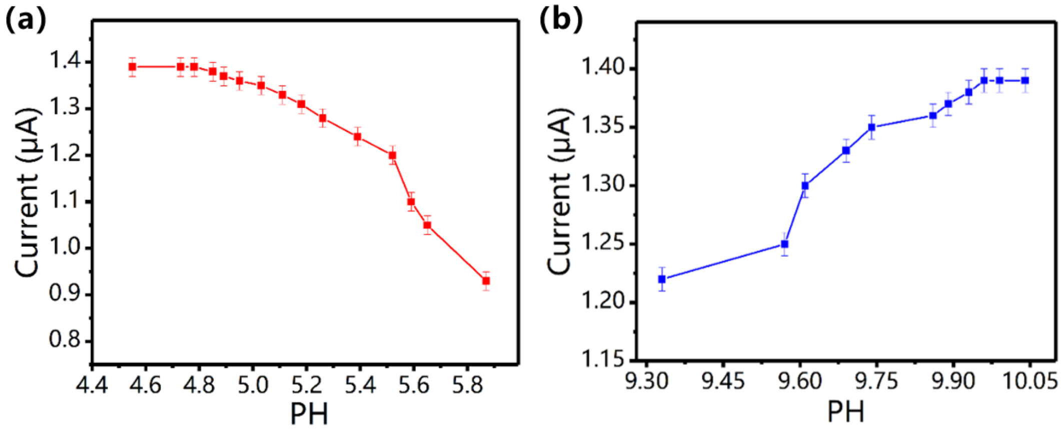

Supplementary Fig. 19 Effect of the PH on the peak value of the current. Variation of the peak value of the receiving current underwater with the PH of **a** acid and **b** alkali solution. Error bars indicate standard deviations.

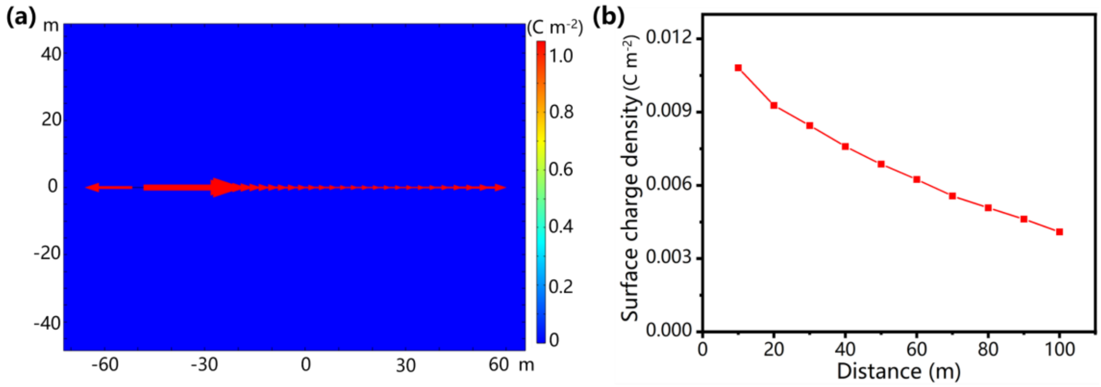

Supplementary Fig. 20 Distribution of the polarization electric field in the water pipe. **a** Distribution of the polarization electric field in the water pipe when the two electrodes is100 m apart. **b** Variation of terminal charges at the receiving electrode with the distance between two electrodes.

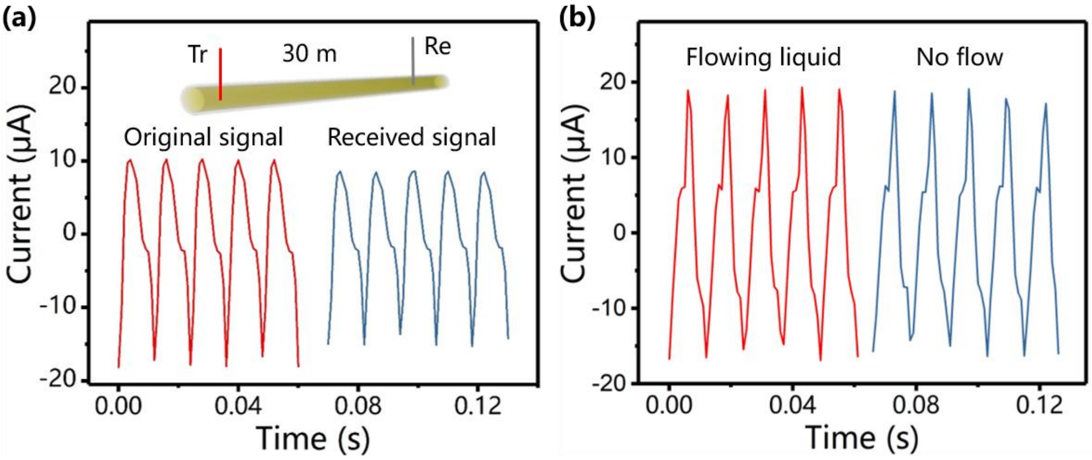

Supplementary Fig. 21 Short-circuit current output of the TENG and received signals in water. **a** Short-circuit current output of the TENG and received signals in the oil and water pipe. Tr represents the transmitting electrode and Re represents the receiving electrode. **b** Received signals in flowing and still liquid.

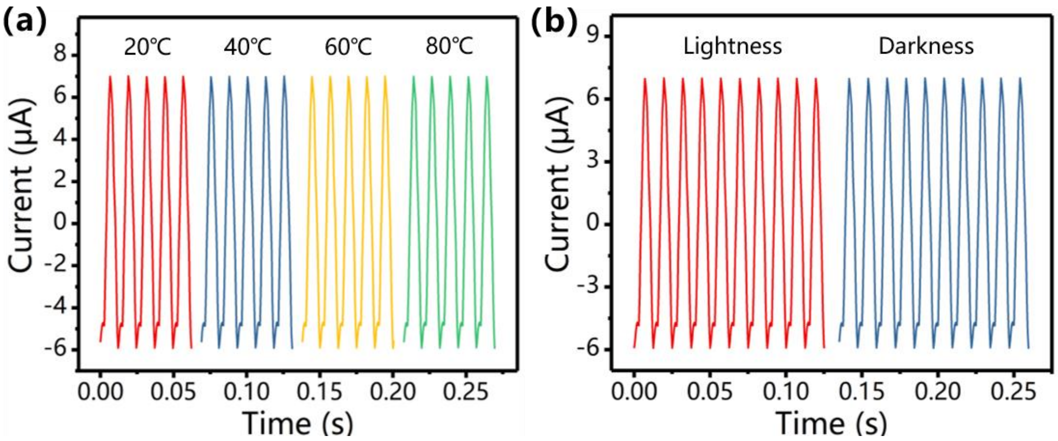

Supplementary Fig. 22 The received current signals under different temperatures and light conditions. The received current signals under different a temperatures b light conditions.

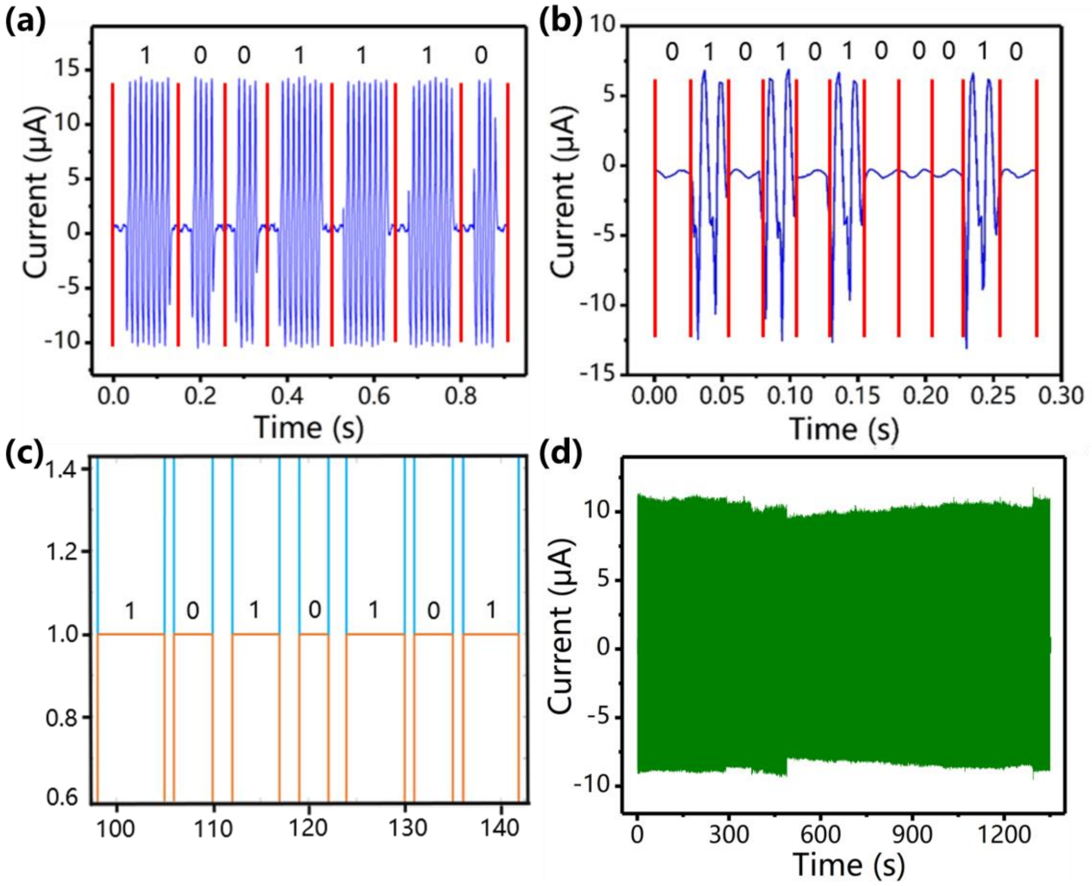

Supplementary Fig. 23 The modulation current signals for data transmission in water. **a** The modulated digital signals with a lower frequency. **b** The modulated digital signals with the setting of “0” and “1” according to the presence or absence of current signals. **c** The digital signals demodulated by the MATLAB. **d** The full-time current signals transmitted for an grey Lena image.

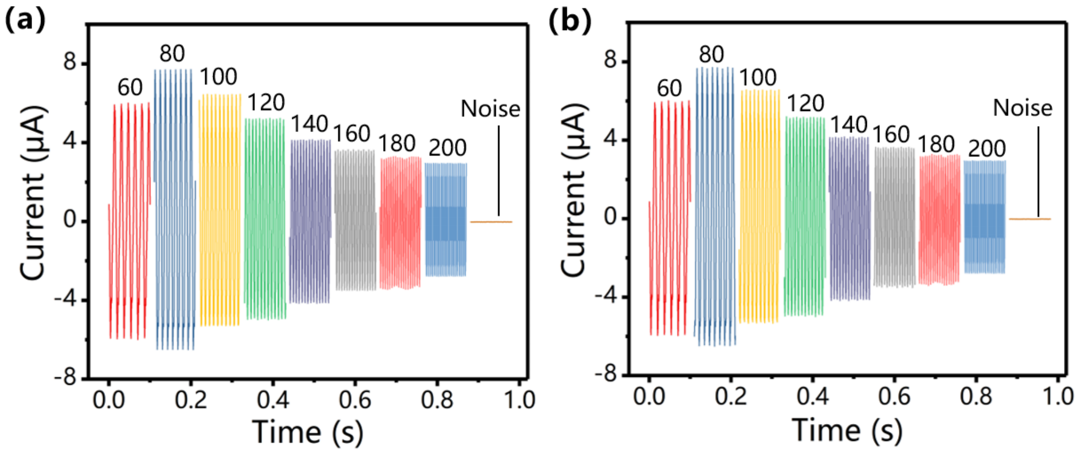

Supplementary Fig. 24 Received current signals in different frequencies. The current signals (a) output by the TENG (b) received underwater at a range of 60-200 Hz.

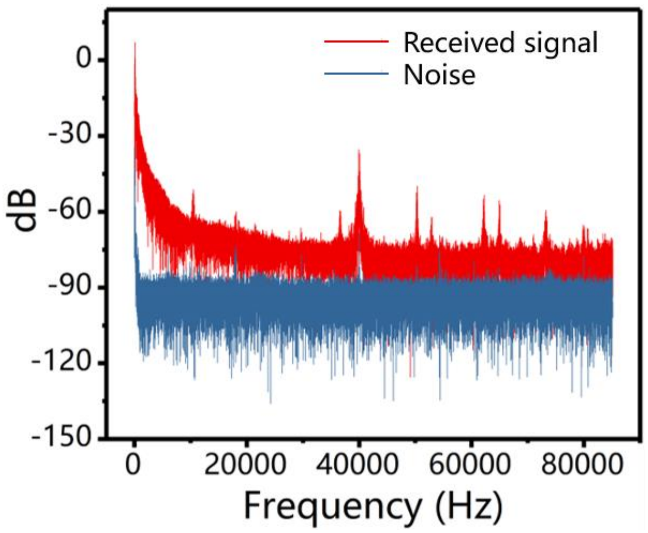

Supplementary Fig. 25 Power spectrum of the modulated digital signals and noise in water.

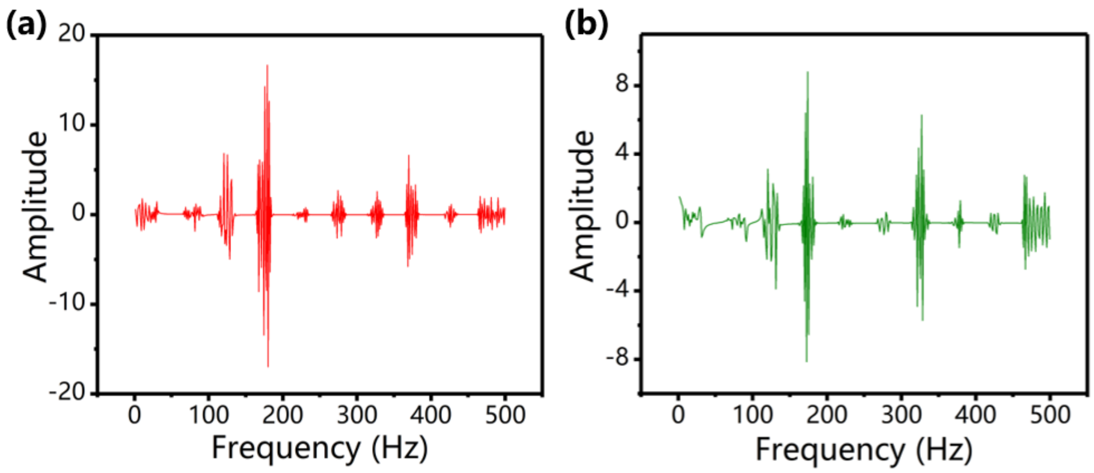

Supplementary Fig. 26 Fourier transform of “red” and “green”. a Fourier transform of “red”. b Fourier transform of “green”.

Supplementary Table 1. Output performance of different types of TENGs.

	Author	Name	Materials	Output	Frequency	Energy	Mode
1	Tiancong Zhao	T-TENG	PTFE balls Nylon film	550 V 5.8 μA	2 Hz	Wave	Free-standing
2	Xiu Xiao	HSI-TENG	PTFE balls Copper film	98 V 3.5 μA	30 Hz	Vibration	Free-standing
3	Hao Wang	S-TENG	PTFE balls Aluminum film	35.98 μA	1 Hz	Wave	Free-standing
4	Yi Xi	TENG	PTFE balls Copper film	170 V 1.43 mA	80 kHz	Ultrasonic wave	Free-standing
5	Chen Chen	μTUD	Silicon membrane Gold	16.7 mV 297 nW	1 MHz	Ultrasonic wave	Contact-separation
6	Venkateswaran Vivekananthan	SE-TENG	silicone elastomer film Ni foam	370 V 6.1 μA	1 Hz	Pressure	Contact-separation
7	Manisha Sahu	MS-HG	BCZT-BH6 Aluminum film	300 V 6.6 μA	1 Hz	Body Activity	Contact-separation
8	Arunkumar Chandrasekhar AMRSC	SP-TENG	PDMS film Paper	70 V 6.5 μA	0.5 Hz	Biomechanical Energy	Contact-separation

Supplementary Table 2. Comparison of acoustic, optical, electromagnetic waves and electric field in underwater communication.

	Acoustic wave	Optical wave	Electromagnetic wave	Electric field
Clear water	Yes	Yes	Yes	Yes
Turbid water	Yes	No	Yes	Yes
Seawater	Yes	Yes	No	Yes
Confined spaces	No	No	Yes	Yes
Obstacles	No	No	Yes	Yes
Water pipes	No	No	Yes	Yes
Ambient light	Yes	No	Yes	Yes
No delay	No	Yes	Yes	Yes
No antenna	Yes	Yes	No	Yes
Self-powered	No	No	No	Yes

Supplementary Notes

Supplementary Note 1. The difference between traditional and TENG based underwater communication approaches.

The working principle of the TENG based underwater communication is different from that of traditional underwater electric field communication. For the TENG based underwater communication, both transmitter and receiver are single electrode. But for the traditional underwater electric field communication, both the transmitter and receiver contain a pair of electrodes[1] (Fig.N1). In order to extend the communication range of this communication system, the power amplifier is adopted to amplify the signals on the transmitting electrodes[2]. And the amplified signals would construct the electric field and form the return current around the transmitter with the changing of the transmitting data. A pair of sensitive electric electrodes can capture electric potential difference from the electric field built by the transmitting electrodes. This approach also doesn't need the antenna[3]. Another difference is that the TENG is self-powered, which can convert mechanical energy into electrical signals[4]. In this way, a self-powered and portable device for underwater communication can be constructed. While the traditional electric field communication must rely on high-power traditional power supply.

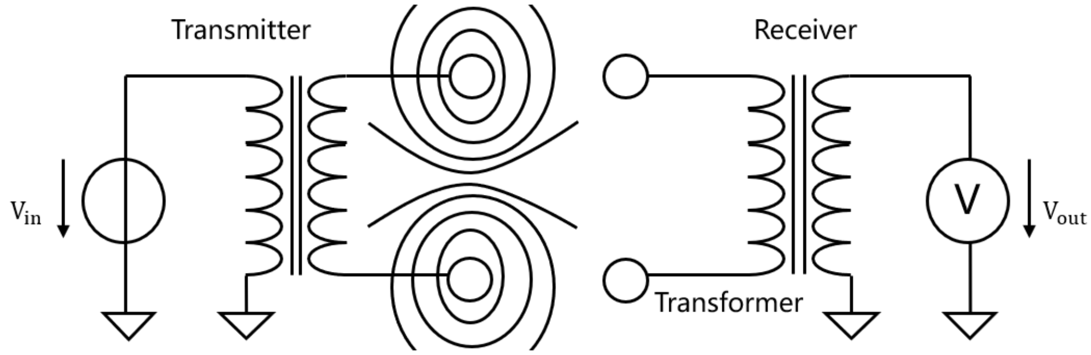

Fig. N1 Schematic diagram of the electric field communication that generated from a pair of electric dipoles.

Supplementary Note 2. The working principle of the TENG via Maxwell's displacement current.

In the Maxwell's displacement current, the first term $\varepsilon_0\partial\mathbf{E}/\partial t$ corresponds to electromagnetic wave, which is the foundation of wireless communication, radar and the concurrent information technology. Studies from Zhonglin Wang indicate that the second term $\partial\mathbf{P}/\partial t$ in the Maxwell's displacement current is directly related to the output electric current of the triboelectric nanogenerator[5,6].

The Maxwell's displacement current is defined as

$$\mathbf{J_D} = \frac{\partial\mathbf{D}}{\partial t} = \varepsilon_0\frac{\partial\mathbf{E}}{\partial t} + \frac{\partial\mathbf{P}}{\partial t}$$

The displacement current is not an electric current formed by moving free charges, but a time-varying electric field (vacuum or media), plus a contribution from the slight motion of charges bound in atoms, dielectric polarization in materials[7,8].

For a contact-separation mode TENG with two dielectrics with permittivity of ε_1 and ε_2 and thicknesses d_1 and d_2, respectively, once the two dielectrics are driven to make physical contact, electrostatic charges are transferred to the surfaces of the two owing to the contact electrification effect. The electrostatic field built by the triboelectric charges (surface charge density σ_c) drives electrons to flow through the external load, resulting in an accumulation of free electrons in the electrode $\sigma_1(z,t)$, which is a function of the gap distance $z(t)$ between the two dielectrics. This is the process of converting mechanical energy into electricity. And the corresponding displacement current density is

$$\mathbf{J_D} = \frac{\partial\mathbf{D}}{\partial t} = \frac{\partial\mathbf{P}}{\partial t} = \frac{\partial\sigma_1(z,t)}{\partial t} = \sigma_c\frac{dz}{dt}\frac{d_1\varepsilon_0/\varepsilon_1 + d_2\varepsilon_0/\varepsilon_2}{(d_1\varepsilon_0/\varepsilon_1 + d_2\varepsilon_0/\varepsilon_2 + z)^2}$$

This equation means that the internal circuit in the TENG is dominated by the displacement current, and the observed current in the external circuit is the capacitive conduction current. The internal circuit and external circuit can meet at the two electrodes, forming a complete loop. Therefore, the displacement current is the intrinsic

physical core of current generation and it is the internal driving force, and the capacitive conduction current in an external circuit is the external manifestation of displacement current.

The difference between the electromagnetic wave and polarization electric field is that the propagation of the electromagnetic wave does not require a medium. When electromagnetic waves propagate in water, they will be largely absorbed by water, especially in seawater with considerable salinity[9]. While a medium required for the propagation of the polarization electric field, the salinity of water is beneficial to the propagation of the polarization electric field. What’s more, the transmission and reception of electromagnetic waves require an antenna, especially the propagation of low-frequency electromagnetic waves requires an antenna of several kilometers, while the propagation of the polarization electric field does not require this.

Supplementary Note 3. The experiment in vacuum.

The experiment is conducted with the transmitting and receiving electrodes all in vacuum (the vacuum bottle is pumped to a vacuum pressure of about -0.093 MPa by a vacuum pump). The result is that the system can't work in vacuum (Fig. N2). According to Maxwell's displacement current theory $\frac{\partial \mathbf{D}}{\partial t} = \varepsilon_0 \frac{\partial \mathbf{E}}{\partial t} + \frac{\partial \mathbf{P}}{\partial t}$, the propagation of the electric field ($\varepsilon_0 \partial \mathbf{E} / \partial t$) doesn't need a medium, while the polarization displacement current based on the second term $\partial \mathbf{P}/\partial t$ only appears in the media. As explained in the manuscript, comparing the TENG-based underwater electric field, the propagation of electromagnetic waves does not require a medium, and the propagation effect is best in vacuum. At this time, $\partial \mathbf{E}/\partial t$ reaches the maximum value, and $\partial \mathbf{P}/\partial t$ is 0. In contrast, the propagation of the polarization electric field requires a medium. As $\partial \mathbf{E}/\partial t$ gets significantly reduced in water, the propagation effect of $\partial \mathbf{P}/\partial t$ gets improved. If the voltage of the TENG is high enough to generate a strong electric field, this electric field can be transmitted and detected in vacuum like electromagnetic waves. In this study, the voltage of the HR-TENG is low (below 30 V), and there are no well-designed antennas for the transmission of the electric field. Therefore, the system can work in water but not in vacuum.

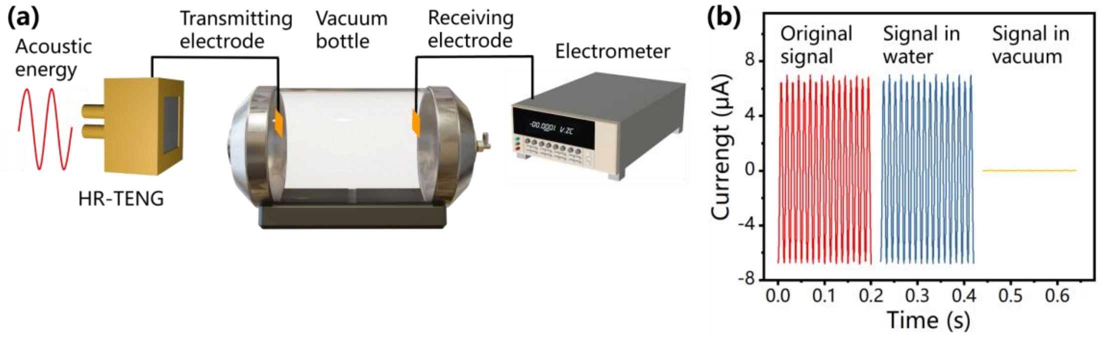

Fig. N2 The experiment in vacuum. **a** Schematic diagram of the experiment. **b** Comparison of the current signals received in different conditions.

Supplementary Note 4. The structure of the HR-TENG.

The structure of the HR-TENG is shown in Fig. 2a. The HR-TENG consists of a Helmholtz resonant cavity, an aluminum film with evenly distributed acoustic holes, and an FEP film with a conductive ink-printed electrode. The Helmholtz resonant cavity is a fundamental acoustic resonance system. It can improve the output performance of the HR-TENG by centralizing the acoustic wave energy and amplifying the sound wave. The FEP material has a strong electronegativity and can generate a large amount of charge transfer during its contact with the aluminum electrode. Given that the FEP material is insulated, a conductive ink electrode with a micron thickness is attached to the back side of the FEP film to transfer electrons.

Supplementary Note 5. Working mechanism of the sound-driven HR-TENG.

Response: Supplementary Fig. N4 depicts the working mechanism of the HR-TENG. The wave characteristic of sound propagation will induce periodic changes in the pressure between the FEP film and the aluminum film, causing the FEP film to oscillate periodically. When the FEP film is in contact with the aluminum film, the electron clouds on the surfaces of the two films overlap, and some of the electrons from the aluminum film enter the deeper potential well of the FEP film[10]. Due to the much higher electronegativity of FEP than aluminum, the free electrons on the surface of the aluminum film transfer to the lowest unoccupied molecular orbital (LUMO) of the FEP interface. Namely, the surface of the FEP film becomes negatively charged, while the aluminum film becomes positively charged (Supplementary Fig. N4i). Due to the change in the acoustic pressure difference, the FEP film is separated from the aluminum electrode. Since the negative and positive triboelectric charges no longer coincide on the same plane, a dipole moment and an electrical potential are generated between the two surfaces. As a result, free electrons are driven to flow from the conductive ink electrode to the aluminum electrode through an external circuit to balance the local electric field[11], thereby producing a positive charge on the conductive ink electrode (Supplementary Fig. N4ii). The flow of electrons lasts until the separation of the two contact surfaces is maximized. Thereafter, the FEP film is pushed back towards the aluminum film by the action of the sound wave (Supplementary Fig. N4iii). The potential drop is weakened at this stage, and free electrons in the aluminum film flow back to the conductive ink electrode through the external circuit (Supplementary Fig. N4iv). Finally, the two surfaces of the FEP film and the aluminum film are parallel again, with the electrical charge distribution returning to its initial state (Supplementary Fig. N4i). At this point, a full cycle of electricity generation has been completed. Subsequently, the FEP film is separated from the aluminum film again due to the propagation of the sound wave, and another cycle of power generation begins.

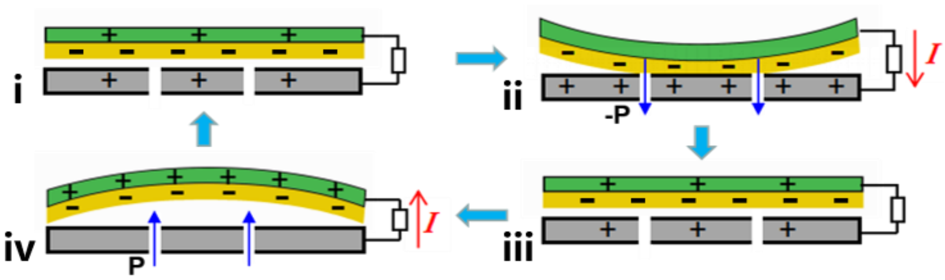

Supplementary Fig. N3 Working mechanism of the sound-driven HR-TENG.

Supplementary Note 6. The analysis of theories and experiments for the whole system.

In stimulating the built-in electric field of TENG, charge accumulates on the transmitting electrode, thereby generating a radiation electric field. Driven by the electric field, the carriers in the water (the charge density is q) move to transfer the charge from the transmitting electrode to the receiving electrode. While electrons transfer between the TENG and the transmitting electrode as well as between the receiving electrode and the electrometer, there is hardly any electron transfer between the two electrodes through water. During the propagation of the electric field, water cannot be electrolyzed, that is, there is no production of hydrogen and oxygen (measured by a combustible gas sensor). After insulating the transmitting electrode by a Kapton tape, the receiving electrode can still receive the current signals transmitted by the TENG (Supplementary Fig. 5).

Therefore, for the whole system, it is more proper for the current in water to be regarded as the displacement current. Under the stimulation of the TENG, a radiation electric field is generated on the transmitting electrode, and water acts as a medium for propagating the electric field. The movement of ions in the water generates a polarization electric field **P**, which causes induced charges on the receiving electrode (charge density is σ), so the signals transmitted by the TENG can be measured by the electrometer.

Maxwell's equations show that both the conduction current and the displacement current will generate magnetic fields, i.e.

$$\nabla \times \mathbf{H} = \mathbf{J} + \frac{\partial \mathbf{D}}{\partial t}$$

Where **H** is the magnetic field strength, **J** is the conduction current density, and **D** is the electric displacement vector. According to the capacitance model

$$\nabla \times \mathbf{H} = \frac{\partial \mathbf{P}}{\partial t} = \frac{\partial \sigma}{\partial t} = \frac{\partial q}{\partial t}$$

Regardless of whether the movement of ions is regarded as conduction current or displacement current, it has the same magnetic effect. The conduction current is generally the direct electron transfer, and the displacement current is generally the

radiation of the electric field to the surrounding space. In the electric field communication process, electrons are not directly transferred from the TENG to the receiving electrode, instead, the electric field is radiated into the surrounding space by the transmitting electrode.

From another perspective, if water can be regarded as a wire, the voltage received by the receiving electrode in the water should be equal to the voltage outputted by the TENG. While the received voltage attenuates by more than one hundred times compared to the original voltage of the TENG, which indicates that water cannot be regarded as a simple wire to the system. This is because the TENG radiates energy as well as the electric field, and this energy can be dissipated in the environment, which also shows that the propagation of underwater electric field has the characteristic of displacement current.

Supplementary Note 7. The effect of the ground on the underwater electric field communication.

A better understanding of “grounding” can be achieved from the following two series of studies (both experimental and numerical), which are both performed in small scale water tank. When the water is grounded, the output current is significantly depressed, but temporal features of electric signals remain consistent with the original signal (Supplementary Fig. 4).

To investigate the effect of the ground on the electric field more clearly, a metal water tank with a size of 1.2 m × 0.2 m × 0.2 m is used, and the water tank is grounded (Supplementary Fig. N5a). Under this condition, the distance of the underwater electric field communication will be very short because the grounded water tank is capable of discharging infinite amount of charge. A rotary TENG with an output voltage of 1000 V and an output current of 175 μA is used in the experiment. When the transmitting electrode is 1 cm away from the receiving electrode, the received current is about 80.5 μA (Supplementary Fig. N5b). As the distance between the two electrodes increases, the current received underwater attenuates quickly, but the rate of the attenuation decreases gradually. When the two electrodes are 15 cm from each other, the current received underwater is about 2.7 μA.

Supplementary Fig. N5c-d show the simulation results of the distribution of polarization electric field. When the bottom of the tank is grounded, the distribution of polarization electric field changes and a part of the electric field lines point to the ground. This means that the contact between water and ground is unfavorable for receiving signals. As the distance between ground and the transmitting and receiving electrodes decreases, the accessible distance for the underwater electric field communication becomes shorter. Conversely, a higher efficiency can be obtained when the ground is located further from the two electrodes.

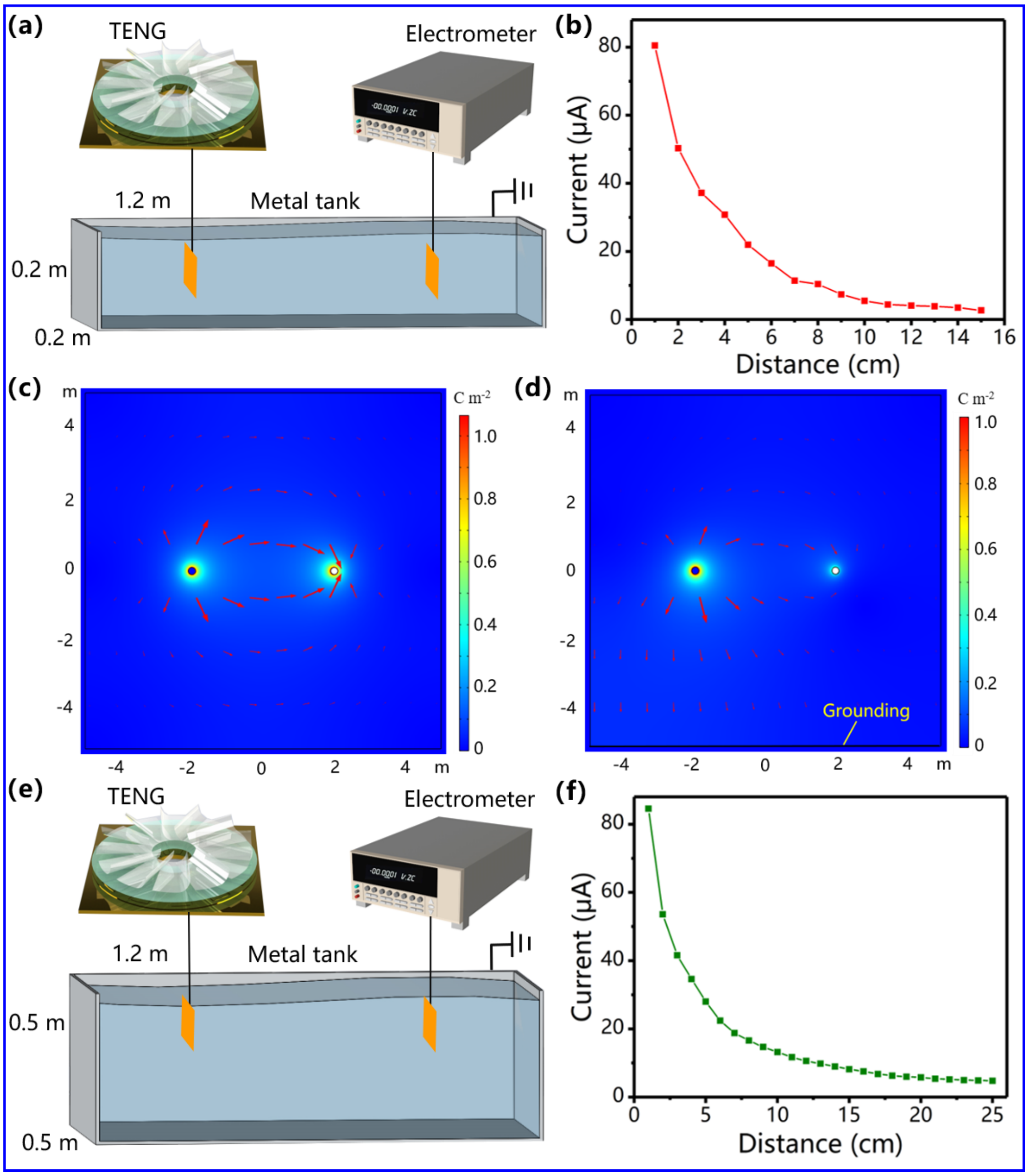

Supplementary Fig. N4 Underwater communication with the water grounded. **a** Schematic diagram of the experiment in a grounded metal water tank with a size of 1.2 m × 0.2 m × 0.2 m. **b** Variation of the peak values of the received current signals with distance. Simulation diagram of the distribution of polarization electric field **c** without and **d** with the water grounded. **e** Schematic diagram of the experiment in a grounded metal water tank with a size of 1.2 m × 0.5 m × 0.5 m. **f** Variation of the peak values of the received current signals with distance.

Supplementary Fig. N5e-f show the underwater electric field communication in a grounded water tank with a dimension of 1.2 m × 0.5 m × 0.5 m, which is slightly larger

than the previous one. When the transmitting electrode is 1 cm away from the receiving electrode, the received current is about 84.6 μA. When the distance between the two electrodes is the same, the current received underwater is always higher than that obtained in the previous experiment. And when the two electrodes are 25 cm apart, the current received underwater is about 4.8 μA, which is even larger than that obtained in the previous experiment at a distance of 15 cm. So the attenuation rate of the current received underwater with distance has been reduced as the water volume increases.

Supplementary Fig. N6 is the simulation of the electric field communication in different water areas when the surrounding water contacts ground. Figure R13a shows the distribution of the polarization electric field in a 1.2 m × 0.2 m × 0.2 m water tank, which corresponds to the experiment shown in Supplementary Fig. N5a. When the distance between the transmitting and receiving electrodes increases from 1 cm to 15 cm, the charge on the receiving electrode decreases from 1.97×10^{-3} C to 5.60×10^{-5} C (Supplementary Fig. N6b). While in the 1.2 m × 0.5 m × 0.5 m water tank (Supplementary Fig. N6c), when the distance between the two electrodes increases from 1 cm to 25 cm, the charge on the receiving electrode decreases from 20.72×10^{-3} C to 5.74×10^{-5} C (Supplementary Fig. N6d). So the same result can be obtained in the simulation that when the surrounding water is grounded, the efficiency of the electric field communication increases as the water volume increases.

The electric field communication in a 1000 m × 1000 m water area (determined based on the capacity of our simulation platform) is simulated, part of which is shown in Supplementary Fig. N5e. When the distance between the two electrodes increases from 1 m to 15 m, the charge on the receiving electrode decreases from 0.1 C to 0.057 C (Supplementary Fig. N6f), in which the attenuation rate of the underwater electric field reduces significantly.

Although the electric field can be depressed by the ground, underwater communication doesn't necessarily happen near the ground. It is necessary to study, develop and optimize communication methods in various underwater scenarios. Actually, the TENGs used in the study are of micro power (only microwatt-level), which is about 4

order of magnitude smaller than the watt-level transmission power of traditional underwater communication equipment. The improvement of the TENG technique and the networking design could greatly increase the power, meanwhile providing the potential for becoming an effective underwater communication alternative.

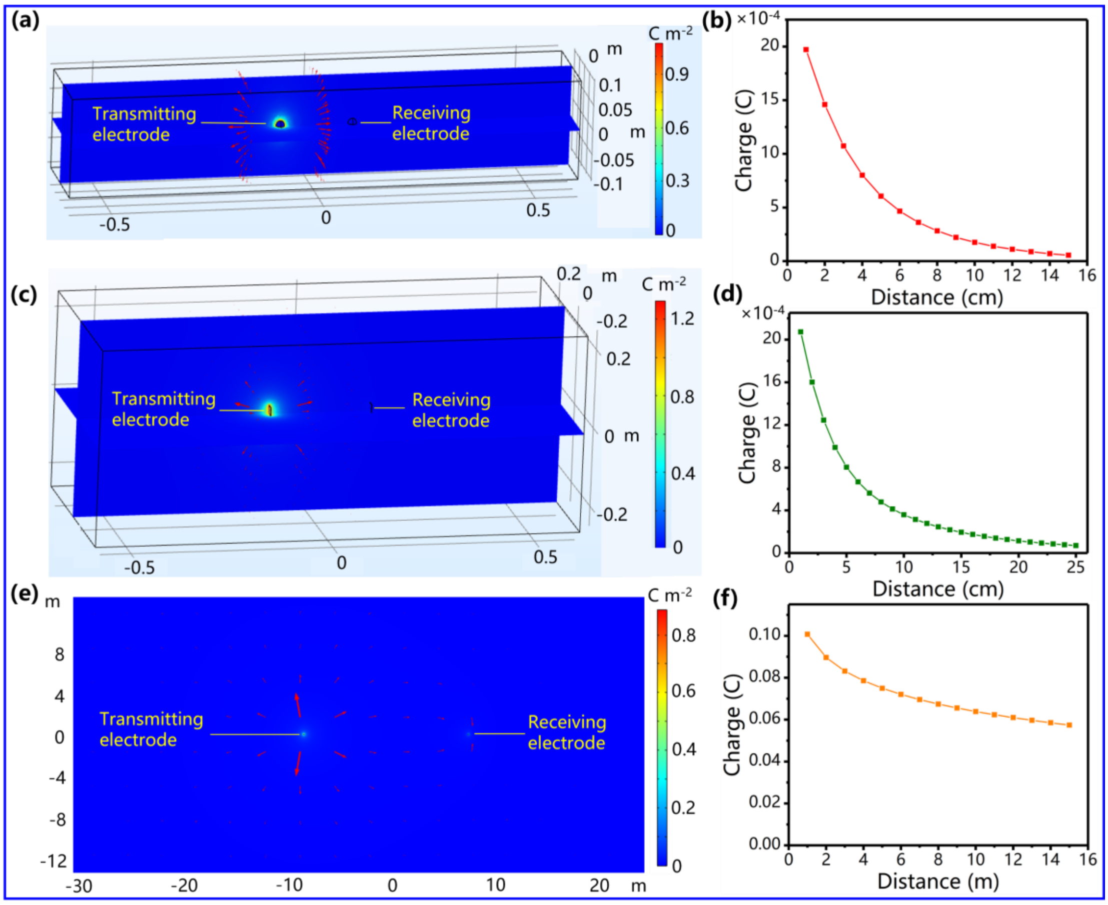

Supplementary Fig. N5 The simulation of different water areas when the surrounding water is in contact with the ground. **a** The distribution of the polarization electric field and **b** variation of the charge on the receiving electrode with distance in a 1.2 m × 0.2 m × 0.2 m water tank. **c** The distribution of the polarization electric field and **d** variation of the charge on the receiving electrode with distance in a 1.2 m × 0.5 m × 0.5 m water tank. **e** The distribution of the polarization electric field and **f** variation of the charge on the receiving electrode with distance in a 1000 m × 1000 m water area.

Supplementary Note 8. The TENG-based underwater communication in high frequencies.

The electric output of the TENG is driven by the built-in electric field[12]. In order to simulate the electric signals output by the TENG at a higher frequency, an external alternating current (16 kHz, about 2 V, 10 mA) is applied on the dielectric material's electrode to affect the built-in electric field, so that the electric output of the TENG can be changed (Fig. N7a). The working frequency of the TENG is 80 Hz, and after the electric signal transmitting through the water, the received signal from the receiving electrode (3 m away from the transmitting electrode) is shown in Fig. N7b. Zooming in on the details of the signal, the waveform is shown in Fig. N7c, demonstrating that there are more high frequency signals. The Fourier transform (Fig. N7d) shows the signal from Fig. N7b consists of low frequency signals (80 Hz dominant frequency and other disturbances) and a 16 kHz higher frequency signal. Furtherly, when an external alternating current with a frequency of 2.7 MHz is applied on the electrode of the dielectric material of the TENG, the underwater electric signal received by an oscilloscope (the maximum sampling frequency of the electrometer is 170 kHz) is shown in Fig. N7e, indicating that the electric field communication approach can work in high frequencies.

An external alternating current with a frequency of 16 kHz is applied on the dielectric material's electrode. After modulating by on-off keying method (2 kHz), the electric signals output by the TENG are transmitted to the water by the transmitting electrode. The signals can be received underwater by the electrometer via the receiving electrode (3 meters away from the transmitting electrode) with a sampling rate of 160 kHz. After filtering out low frequencies (80 Hz and other interference), the complete signals and part of the current signals are shown in Fig. N8 a-b, and the picture can be got by demodulating the signals. This experiment has been repeated 10 times with only two wrong data, demonstrating good accuracy.

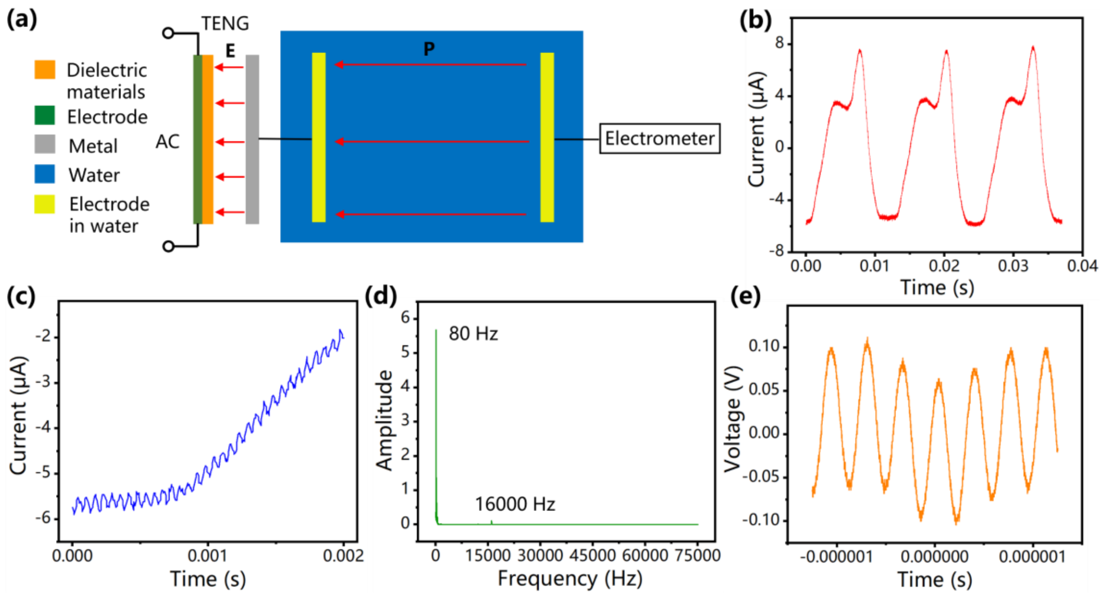

Fig. N6 The TENG-based underwater communication in high frequencies. **a** Schematic diagram of the experiment. **E** represents built-in electric field of the TENG, and **P** represents underwater polarization electric field. When the external alternating current is 16 kHz, the current signal received underwater in **b** 0.04 s and **c** 0.002 s. **d** The Fourier transform of the current signal received underwater **e**. The signal received underwater by an oscilloscope when the external alternating current is 2.7 MHz.

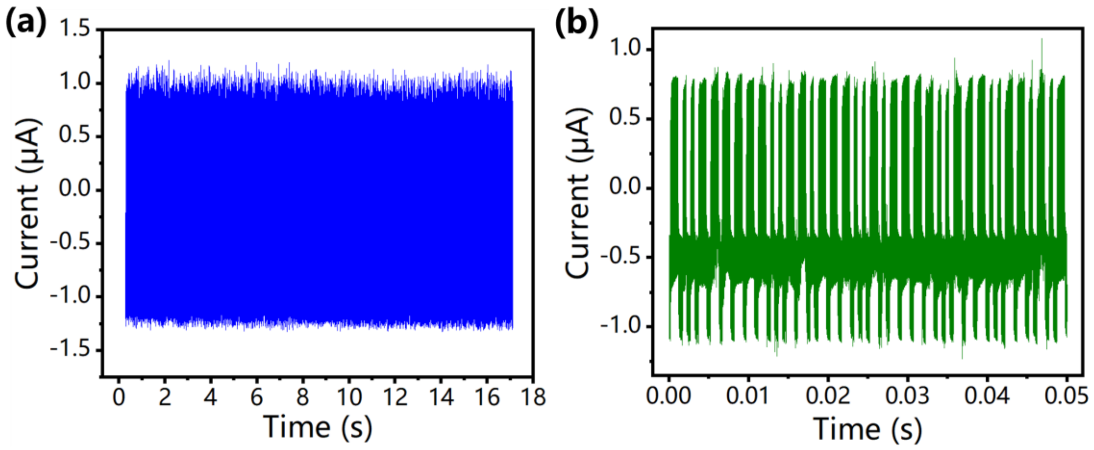

Fig. N7 The modulated signals transmitted for an image. **a** Complete and **b** part of current signals received underwater for a gray Lena image.

Supplementary Note 9. The working principle of the auxiliary circuit.

In order to demonstrate the application prospect of this communication method, a power management circuit was constructed to improve the output current which can decrease the voltage from a few thousand volts to hundreds of volts and generate a stronger current peak signal. This current signal can transfer though the water and be acquired by a well-designed current acquisition board. This current acquisition board with pA resolution can acquire the instruction from the transmitting electrode and control the movement of underwater robots by program analyzing.

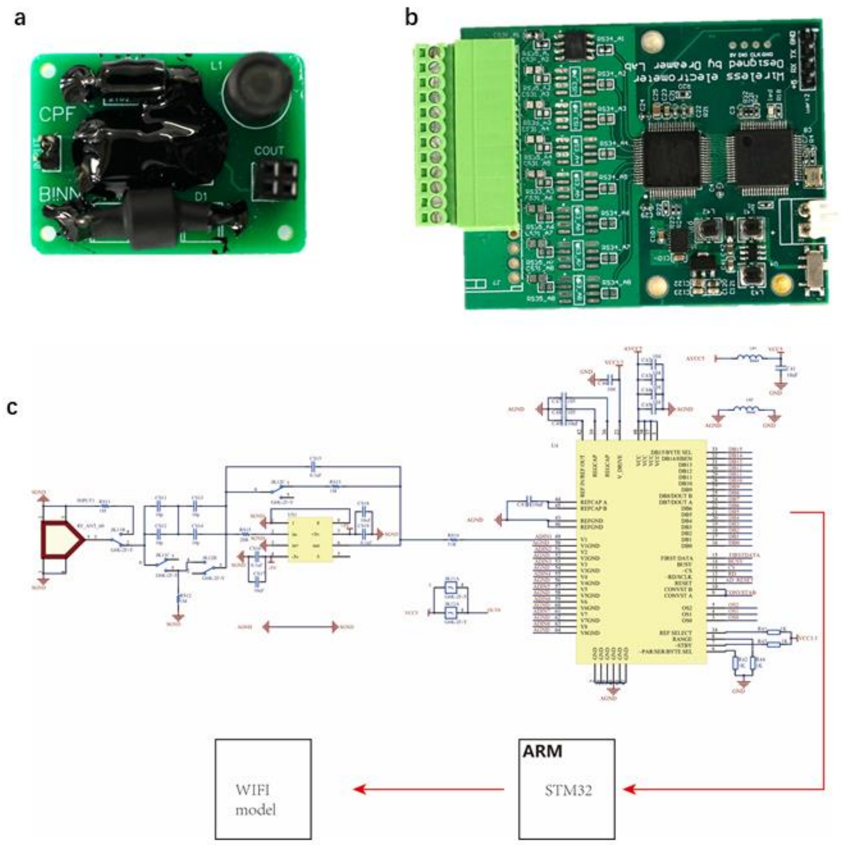

Fig. N8 The photograph of a power management circuit as well as b current signal acquisition board and c the circuit diagram.

Supplementary References

1. Esemann, T., Ardelt, G. & Hellbrück, H. Underwater Electric Field Communication. in *Proceedings of the International Conference on Underwater Networks & Systems - WUWNET '14* (Rome, Italy, 2014).
2. Momma, H. & Tsuchiya, T. Underwater Communication by Electric Current. in *OCEANS '76.* (Washington, USA, 1976).
3. Zoksimovski, A., Sexton, D., Stojanovic, M. & Rappaport, C. Underwater electromagnetic communications using conduction – Channel characterization. *Ad Hoc Networks* **34**, 42-51 (2015).
4. Yu, J. *et al.* Contact-electrification-activated artificial afferents at femtojoule energy. *Nature communications* **12**, 1581 (2021).
5. Sun, M., Lu, Q., Wang, Z. L. & Huang, B. Understanding contact electrification at liquid-solid interfaces from surface electronic structure. *Nature communications* **12**, 1752 (2021).
6. Luo, J., Gao, W. & Wang, Z. L. The Triboelectric Nanogenerator as an Innovative Technology toward Intelligent Sports. *Advanced materials* **33**, e2004178 (2021).
7. Zi, Y. *et al.* Field Emission of Electrons Powered by a Triboelectric Nanogenerator. *Advanced Functional Materials* **28**, 1800610 (2018).
8. Chen, C. *et al.* A method for quantitatively separating the piezoelectric component from the as-received "Piezoelectric" signal. *Nature communications* **13**, 1391 (2022).
9. Lloret, J., Sendra, S., Ardid, M. & Rodrigues, J. J. Underwater wireless sensor communications in the 2.4 GHz ISM frequency band. *Sensors* **12**, 4237-4264 (2012).
10. Zi, Y. *et al.* Harvesting low-frequency (< 5 Hz) irregular mechanical energy: a possible killer application of triboelectric nanogenerator. *ACS nano* **10**, 4797-4805 (2016).
11. Zi, Y., Wu, C., Ding, W. & Wang, Z. L. Maximized Effective Energy Output of Contact-Separation-Triggered Triboelectric Nanogenerators as Limited by Air

Breakdown. *Advanced Functional Materials* **27**, 1700049 (2017).

12. Wang, Z. L., Lin, L., Chen, J., Niu, S. & Zi, Y. Triboelectric Nanogenerator: Freestanding Triboelectric-Layer Mode. (2016).
