# Supplementary material for: Underwater wireless communication via TENG-generated Maxwell’s displacement current
Source: Nat Commun. 2022 Jun 9;13:3325. doi: 10.1038/s41467-022-31042-8 (PMC9184604; doi:10.1038/s41467-022-31042-8)
Supplement: Supplementary file 2 — Description of Additional Supplementary Files [file 41467_2022_31042_MOESM2_ESM.pdf]

Description of Additional Supplementary Files

File Name: Supplementary Movie 1
Description: Modulation and demodulation of current signals for data transmission in water.

File Name: Supplementary Movie 2
Description: Self-powered communication based on the voice-driven TENG.

File Name: Supplementary Movie 3
Description: Self-powered communication based on the button-type TENG.

File Name: Supplementary Movie 4
Description: Underwater communication in a 50 × 30 × 5 m basin.
